# A problem-oriented systems approach to primary care system development: development and initial testing of the problem-oriented primary care system development record

**DOI:** 10.1186/s12913-020-05581-z

**Published:** 2020-08-01

**Authors:** Ali Rafik Shukor, Erica Barbazza, Niek Klazinga, Dionne Sofia Kringos

**Affiliations:** Department of Public and Occupational Health, Amsterdam UMC, University of Amsterdam, Amsterdam Public Health research institute, Meibergdreef 9, Amsterdam, 1105 AZ The Netherlands

**Keywords:** Primary care system development, Performance assessment, Primary care framework, Problem-oriented record, Health policy

## Abstract

**Background:**

There is significant global policy interest related to enabling a data-driven approach for evidence-based primary care system development. This paper describes the development and initial testing of a prototype tool (the *Problem-Oriented Primary Care System Development Record, or PCSDR*) that enables a data-driven and contextualized approach to primary care system development.

**Methods:**

The PCSDR is an electronic record that enables the systematic input, classification, structuring, storage, processing and analysis of different types of data related to the structure, function and performance of primary care systems over time. Data inputted into the PCSDR was coded using the WHO’s PHC-IMPACT framework and classification system. The PCSDR’s functionalities were tested by using a case study of primary care system development in Tajikistan.

**Results:**

Tajikistan’s case study demonstrated that the PCSDR is a potentially effective and conceptually-sound tool for the input, classification, structuring and storage of different data types from myriad sources. The PCSDR is therefore a basic data entry and data management system that enables query and analytics functions for health services research and evidence-based primary care system development functions.

**Conclusions:**

The PCSDR is a data system that enables a contextualized approach to evidence-based primary care system development. It represents a coherent and effective synthesis of the fields of primary care system development and performance assessment. The PCSDR enables analysts to leverage primary care performance assessment frameworks for a broad range of functions related to health systems analysis, improvement and the development of learning health systems.

## Background

The development and realization of effective, efficient and equitable health care systems is contingent on policy-makers applying evidence-based interventions [1]. The WHO’s new Division of Data, Analytics and Delivery aims to enable evidence-based development functions by “significantly enhancing the collection, storage, analysis and usage of data to drive policy change in countries” [2].

The successful operationalization of such a mandate requires the development of robust and conceptually sound data systems [3, 4]. At the most basic level this includes a data system infrastructure that is comprised of a data source system (i.e. a structured record for input and classification of different types of data from myriad sources) and a data management system that enables the structuring, storage and processing of source data (i.e. within a structured relational database), as well as linkage and integration with external databases [3, 5]. Such data systems have the potential to enable powerful health system analytics, modeling and reporting functions, which in turn support research, evaluation and evidence-informed decision-making, ultimately enabling health systems development [3].

To effectively collect, assess and use data for international health care system development, data systems must be conceptually sound (i.e. systems-oriented), content valid and fit-for-purpose. The critical importance of systems-orientation was reaffirmed in the 2019 report *Crossing the Global Quality Chasm: Improving Health Care Worldwide* [6]. Over the past four decades, widespread scientific and policy consensus has emerged on the conceptual basis for orienting health care system development using systems theory [7]. This is manifested through systems-oriented conceptual models such as the WHO ‘Building Blocks’ framework, which is intended to enable health systems strengthening by creating “a taxonomy that would permit clarification of the indicators, data sources and collection methods, and the analytics underpinning monitoring and evaluation” [7]. Such models are underpinned by Dr. Avedis Donabedian’s ‘structure-process-outcome’ classification of health services variables and Prof. Barbara Starfield’s working model for health services research [8, 9].

Other contemporary development-oriented primary care system performance frameworks include the WHO’s *Primary Care Evaluation Tool* (PCET), the *European Tool for Monitoring Impact, Performance and Capacity of Primary Health Care* (PHC-IMPACT) and the *Primary Health Care Performance Initiative* (PHCPI) developed by the Gates Foundation, WHO and the World Bank Group [10–14].

There is significant policy and scientific interest globally in leveraging these performance frameworks to systematically enable evidence-based primary care system development [15]. This paper presents the development and testing of a novel prototype tool that leverages these frameworks for primary care system development. The tool – called the Problem-Oriented Primary Care System Development Record (PCSDR) – aims to support analysts to leverage primary care performance assessment frameworks for contextualized evidence-based primary care system development, by enabling the following key functions:

- collection and entry of different types of data related to the structure, function and performance of primary care systems, and from a myriad of sources *(systematic input);*
- coding of the data using scientific classification systems *(classification, structuring, processing)*;
- measurement of primary care system performance *(analysis)*;
- assessment of determinants of performance *(analysis)*.

**Fig. 1:**
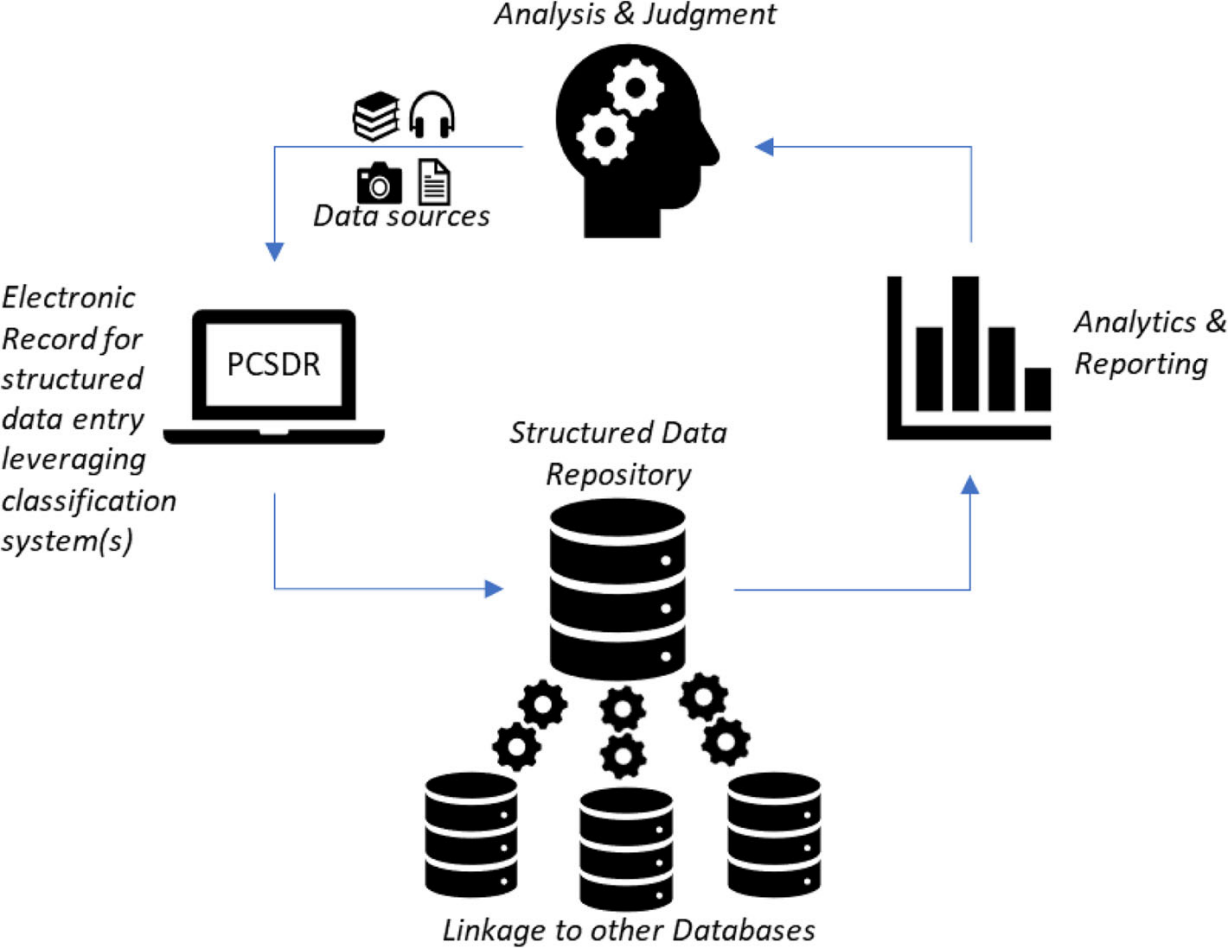
Problem-Oriented Primary Care System Development Record (PCSDR) data system

In essence, the PCSDR is an electronic record that enables the structuring and classification of data pertaining to primary care system development. It enables classification using contemporary primary care system performance frameworks, such as PHC-IMPACT [14]. Similar to electronic records used by other fields, the PCSDR data is structured and stored using a Relational Database Management System. This structured database, which can also be linked to external databases, enables the development of contextualized knowledge that can be used for research and analysis, to support various primary care system development functions such as cross-learning, assessment and audits (Fig. 1). In its ability to store and record information, the PCSDR also presents a means to support the exchange of information – the institutional memory – that is often lost between policy cycles.

This paper describes the PCSDR’s conceptualization and technical development, and uses a case study on Tajikistan’s primary care system to describe and assess the tool’s key functionalities. The paper will discuss how the tool’s functionalities enable an evidence-informed, contextualized and problem-oriented systems approach to primary care system development.

## Methods

The methodology is comprised of six sections, outlining the PCSDR’s key theoretical, methodological and empirical considerations:

- Conceptualization
- Classification system
- Data management system
- Technical development
- Testing: Tajikistan case study
- Analysis of key functionalities

### Conceptualization of the PCSDR

Conceptually, the PCSDR is an electronic record that is biaxial in structure. The horizontal axis adapts a generic problem-oriented record structure consisting of the following key system development functions [F(x)], namely:

- Problem recognition;
- Assessment;
- Intervention; and,
- Follow-up.

These primary care system development functions *[F(x)]* are concrete actions and activities that can influence the sub-systems of primary care (i.e. system domains such as financing, workforce, information technology, etc.).

In turn, these primary sub-systems (denoted by alpha (α)) are conceptualized and classified using the PHC-IMPACT primary care system framework, and comprise the vertical axis of the PCSDR [14].

**Fig. 2:**
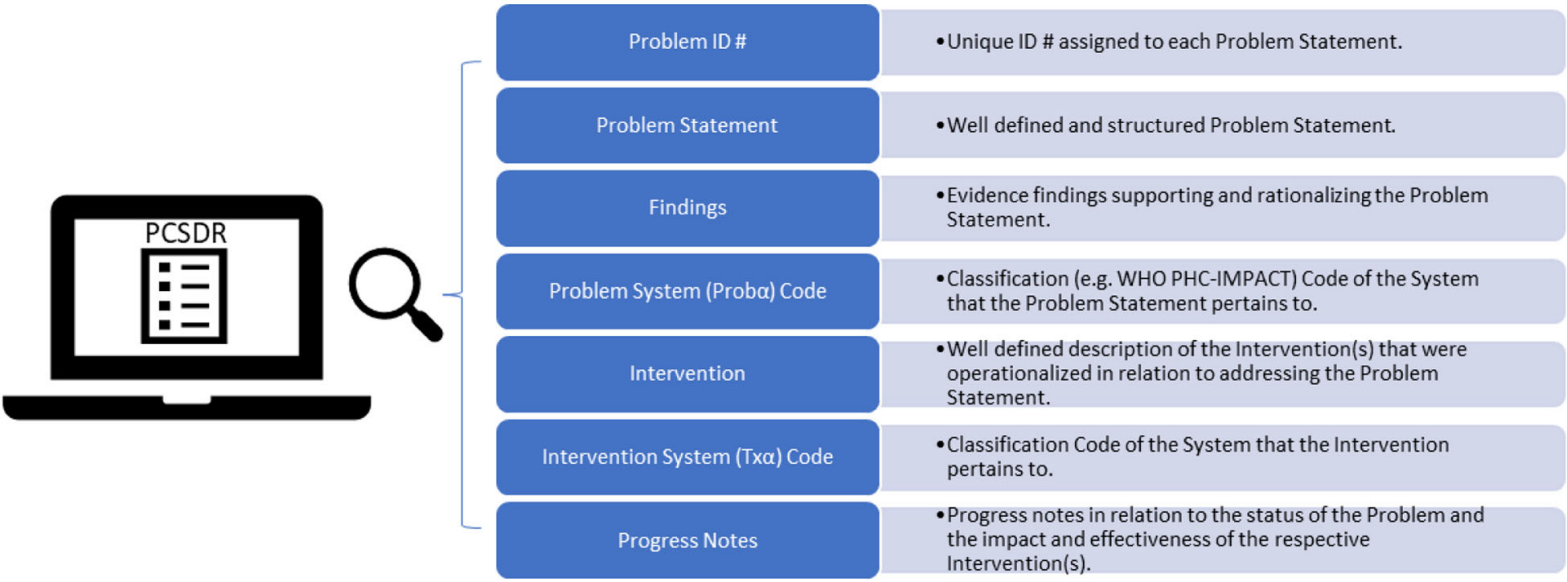
Content of the PCSDR

Both development functions [F(x)] and primary care sub-systems (α) upon which they act are respectively structured and coded using the PCSDR’s seven fields (i.e. Problem Statement, Findings, Problem System Code [Probα Code], Intervention, Intervention System Code [Txα Code], and Progress Notes) (Fig. 2).

The PCSDR is therefore a biaxial electronic record comprised of key development functions [F(x)] (PCSDR x-axis), and the subject matter these functions pertain to (i.e. primary care sub-systems) (PCSDR y-axis). Each PCSDR axis has distinct conceptual requirements: (i) development functions [F(x)] must be *problem-oriented*, and (ii) the classification system of the domain’s subject matter must be *systems-oriented*.

This study is based on the assumption that primary care system development functions *[F(x)]* (i.e. problem recognition, assessment, intervention and follow-up) must be *problem-oriented*.

Problem-orientation enables the PCSDR to structure and contextualize interactions in a way that renders performance states and commensurate development functions meaningful. Problem-orientation is premised on a pragmatist philosophical conception of knowledge as problem-solving, and is one of the principal attributes for the domain of systems-oriented policy science [16–19].

Problem-oriented development functions, in turn, must be premised on influencing the sub-systems of primary care (α), as conceptualized by rigorous systems-oriented frameworks such as the WHO PHC-IMPACT [13, 14].

### PCSDR classification system

**Fig. 3:**
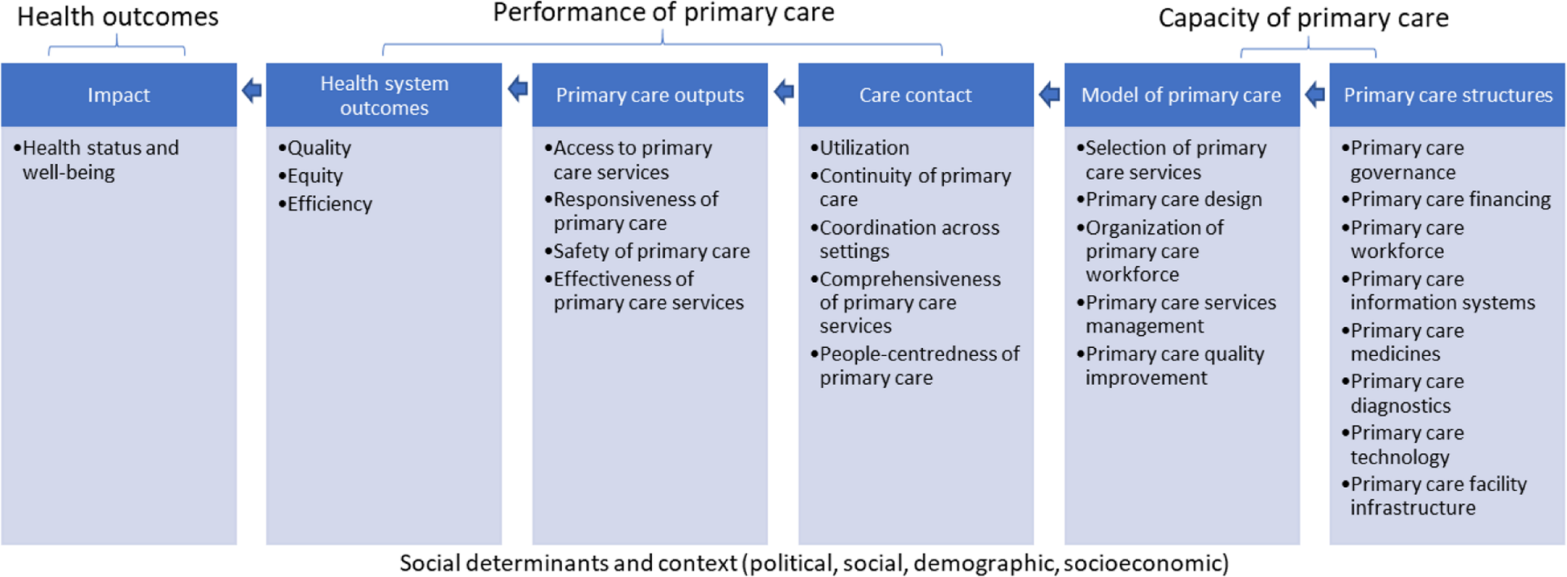
WHO PHC-IMPACT Framework [13, 14]. The bullets in the grey boxes are PHC-IMPACT framework “Dimensions”. Each Dimension is comprised of “Sub-Dimensions”. Each “Sub-Dimension” is comprised of “Indicators”. For example, WHO Dimension “Access to Primary Care Services” is comprised of Sub-dimensions “Accessibility” (code ACS1), “Financial affordability” (code ACS2) and “Acceptability” (code ACS3). Sub-dimension ACS1 is comprised of three Indicators: “Same day appointments” (code ACS1A), “Waiting time” (code ACS1B) and “Provider absence rate” (ACS1C)

The PCSDR enables the scientific coding of problem and intervention systems (α), which are essential for data management, analysis, research and evaluation. The design of the PCSDR is modular and agnostic; therefore, it uses different primary care classification systems, at least for exploratory purposes. The PCSDR’s structure thereby enables flexibility and adaptability, while maintaining rigour. In this study, the PCSDR was coded using the WHO’s PHC-IMPACT framework classification system (Fig. 3) [10, 12–14].

It is important to note that the PHC-IMPACT framework, classification system and indicators are still undergoing development, and its indicator database has not yet been populated [13, 14]. Once the PHC-IMPACT framework and indicator database are finalized and operationalized, it would be possible to link PCSDR codes with performance indicator data.

### The data management system: capturing complexity

The PCSDR is premised on the assumption that primary care system performance is the result of complex interactions of combinations of different systems [17, 20]. The PCSDR was therefore consciously designed to systematically capture, structure and map this complexity. The PCSDR is able to structure and record the content and dynamics of complex interactions between any number of systems, because it is operationalized using a Relational Database Management System.

The structure of the PCSDR enables multi-system variables to be systematically entered, structured and stored into a relational database. Operationalizing the electronic record using a relational database enables data to be structured as knowledge in the form of a semantic network. This approach operationalizes a systems paradigm, whereby knowledge is conceived as a network of interconnections of constituent entities [16, 17]. Knowledge is represented by the network itself, which is quite literally more than the sum of its individual entities [17, 21].

Operationally, the relational database provides a framework that captures statements regarding the content and dynamics of relationships between the PCSDR’s fields (i.e. its classified core content). Furthermore, through data linkage, the database can capture statements regarding relationships between the content of the record’s database and the entities of external databases. The relational database therefore facilitates powerful functions related to structuring, storing, querying and analyzing the content and relationships of data. These functionalities can support scientific research, evaluation and ongoing iterative knowledge generation.

### Technical development

The PCSDR was created using a relational database (MS Access). Two experts in health care informatics provided advice and guidance regarding the design and development of the relational database. The database (Additional File 1) is comprised of the following linked tables:

- the PCSDR table;
- the PCSDR data entry form;
- the WHO PHC-IMPACT classification system; and
- Citations (unlinked table includes MS Word document attachment).

It is important to note that the WHO classification system was originally received as a flat MS Access spreadsheet file. Its data content was converted into normalized relational database files. This conversion is critical for the PCSDR’s basic functionality, to avoid data corruption, offer scalability, and to ensure data and referential integrity.

To illustrate how the integration of external databases can provide additional contextual information, the following tables were included in the relational database:

- public health indicators table using data from the WHO Global Health Observatory [22];
- social, economic and political indicators table using data of United Nations databases [23]; and
- state fragility indicators table according to the Fragile State Index (FSI) and Deutsches Institut für Entwicklungspolitik (DIE) Constellations of State Fragility [24, 25].

### Case study: populating Tajikistan’s PCSDR

Tajikistan was chosen as a case study to test the PCSDR, due to alignment with recent and ongoing WHO primary care system strengthening work, with ample and accessible data availability. Tajikistan’s PCSDR’s content was derived from a scoping literature review, performed by adapting the York approach outlined by Arksey and O’Malley [26].

The search strategy involved several literature sources, including peer-reviewed scientific journals, official reports and secondary documents. Search terms were purposefully kept broad as the goal was to conduct a sensitive rather than specific search.

The first author performed the initial selection and review of relevant literature. Inclusion and exclusion criteria related to whether literature contained meaningful content in relation to primary care system development in Tajikistan, guided by the theoretical primary care system domains outlined by the WHO’s PHC-IMPACT framework.

PubMed was searched using combinations of the terms “Tajikistan”, “primary care”, “primary health care”, “health care” and “healthcare”, for articles published between 1990 and May 2019. The search date was selected as 1991 marking Tajikistan’s independence from the Soviet Union and the beginning of substantive primary care system reforms. A “grey” news and document search (using the keywords “Tajikistan” coupled with “healthcare”, “health care”, “primary care”, “primary health care” along with the framework’s domain keywords) was performed in English using Google. Where possible, website searches were performed in a systematic manner, and site maps, publication links and internal website search engines were leveraged, when available. Snowball searches to identify additional data sources were performed by searching the reference lists of key scientific articles, reports and documents found.

Themes and data from the literature were abstracted, synthesized and organized using thematic content analysis. Findings were compiled and triangulated against the various data sources. Data were cited and hyperlinked to their original online source, when available.

Synthesized findings were shared with the co-authors (NK, DK), who are experienced health system researchers. The co-authors contributed to the analysis, and provided guidance regarding the initiation of additional searches to enable the completeness and validation of findings.

To further complete and validate the data, the PCSDR’s content was exported to MS Word and reviewed by an expert from the WHO, for completeness and accuracy. The expert was requested to pay particular attention to the “Problem Statement” and “Findings” fields, and to provide a brief summary and indicators describing the original problems, going back to year 2000, since they were often poorly articulated or described in the literature. Furthermore, the expert was asked to include any active (i.e. ongoing) problems that have been overlooked, along with related content and/or references. The expert was asked to include any important references that may have been missed or are available in the Tajik language, along with a brief synthesis of their content. The expert’s proposed revisions and comments were reviewed by the first author, who made changes using MS Word’s “Track Changes” feature.

Tajikistan’s PCSDR was coded using WHO’s PHC-IMPACT and PCET classification system. Codes (Probα and Txα) were assigned according to technical criteria of relevance and accuracy. The coding process entailed significant technical judgment and subjectivity, particularly in cases where problem statements, interventions and codes were poorly defined. PCET codes were only assigned to problem systems, since that specific coding exercise was related to demonstrating the potential of cross-mapping classification systems.

### Analysis of functionalities

The MS Access relational database functionality enables various useful data management tasks that support evidence-informed decision-making for the field of primary care system development, such as search and retrieval, structured queries, question answering and definition acquisition [27].

The following queries were performed to demonstrate and analyze the PCSDR’s core functionalities:

- Simple queries of fields, as well as queries of relationships within and between problem and intervention system codes, which enable health services research, performance assessment and development activities.

## Results

The results section reports and describes the content of the PCSDR and its key functionalities, using Tajikistan as a case study.

### PCSDR relational database content

The PCSDR relational database (Additional File 1) is comprised of:

- the WHO PHC-IMPACT classification system table;
- the PCSDR data entry form;
- the PCSDR table (i.e. the record table); and
- contextual tables for Tajikistan.

**Fig. 4:**
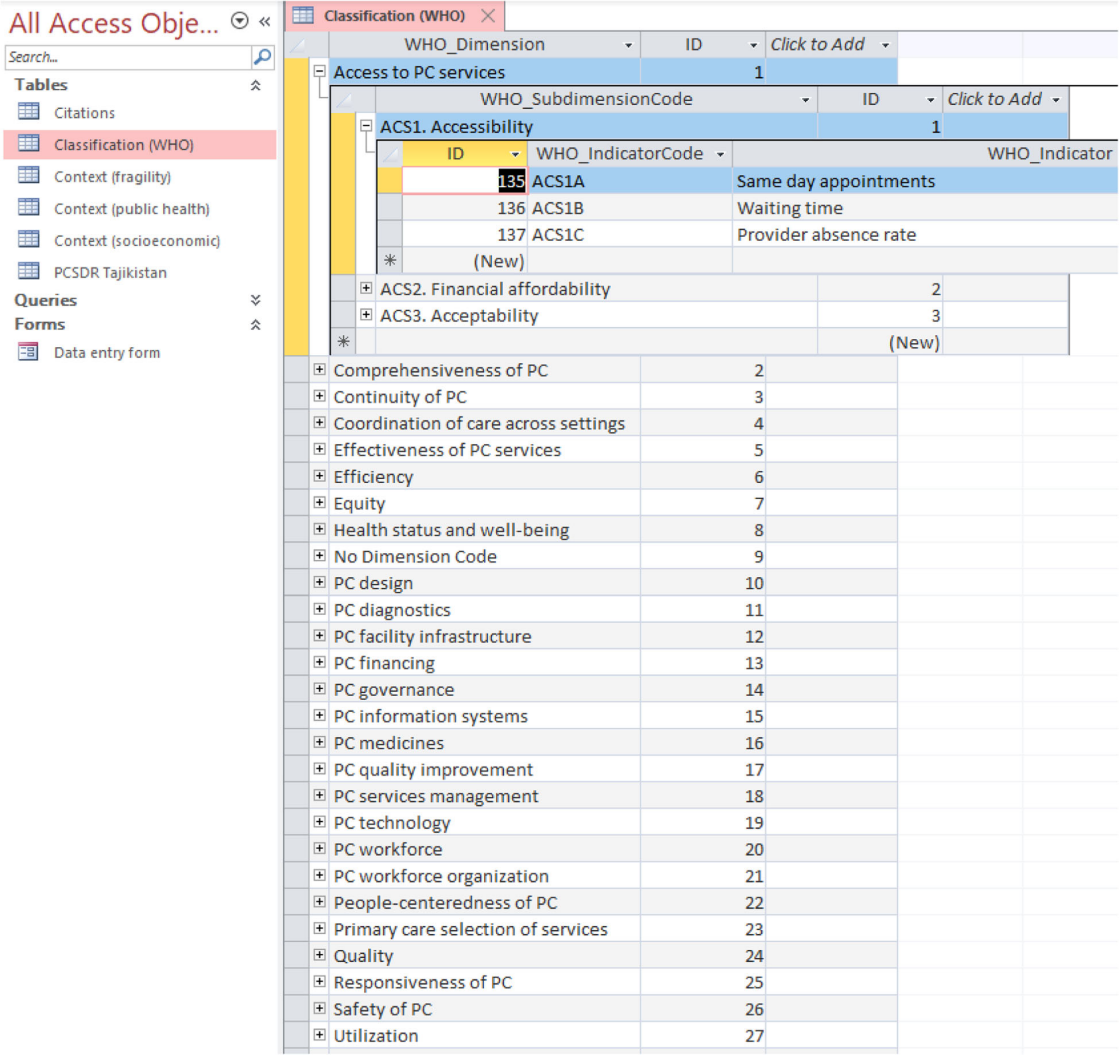
Adapted WHO PHC-IMPACT classification table (screenshot)

The WHO PHC-IMPACT classification was converted to a normalized set of database tables referred to as ‘Dimension’, ‘Sub-dimension’ and ‘Indicator’ relational tables, respectively. This helps avoid data corruption, enable scalability, and ensures data and referential integrity. The ‘Dimension’ database table enables coding of the PCSDR, and acts as an accessible and quick reference (Fig. 4). For example, the WHO Dimension “Access to PC Services” is comprised of Sub-dimensions “Accessibility” (code ACS1), “Financial affordability” (code ACS2) and “Acceptability” (code ACS3). Sub-dimension ACS1 is comprised of three Indicators: “Same day appointments” (code ACS1A), “Waiting time” (code ACS1B) and “Provider absence rate” (ACS1C).

**Fig. 5:**
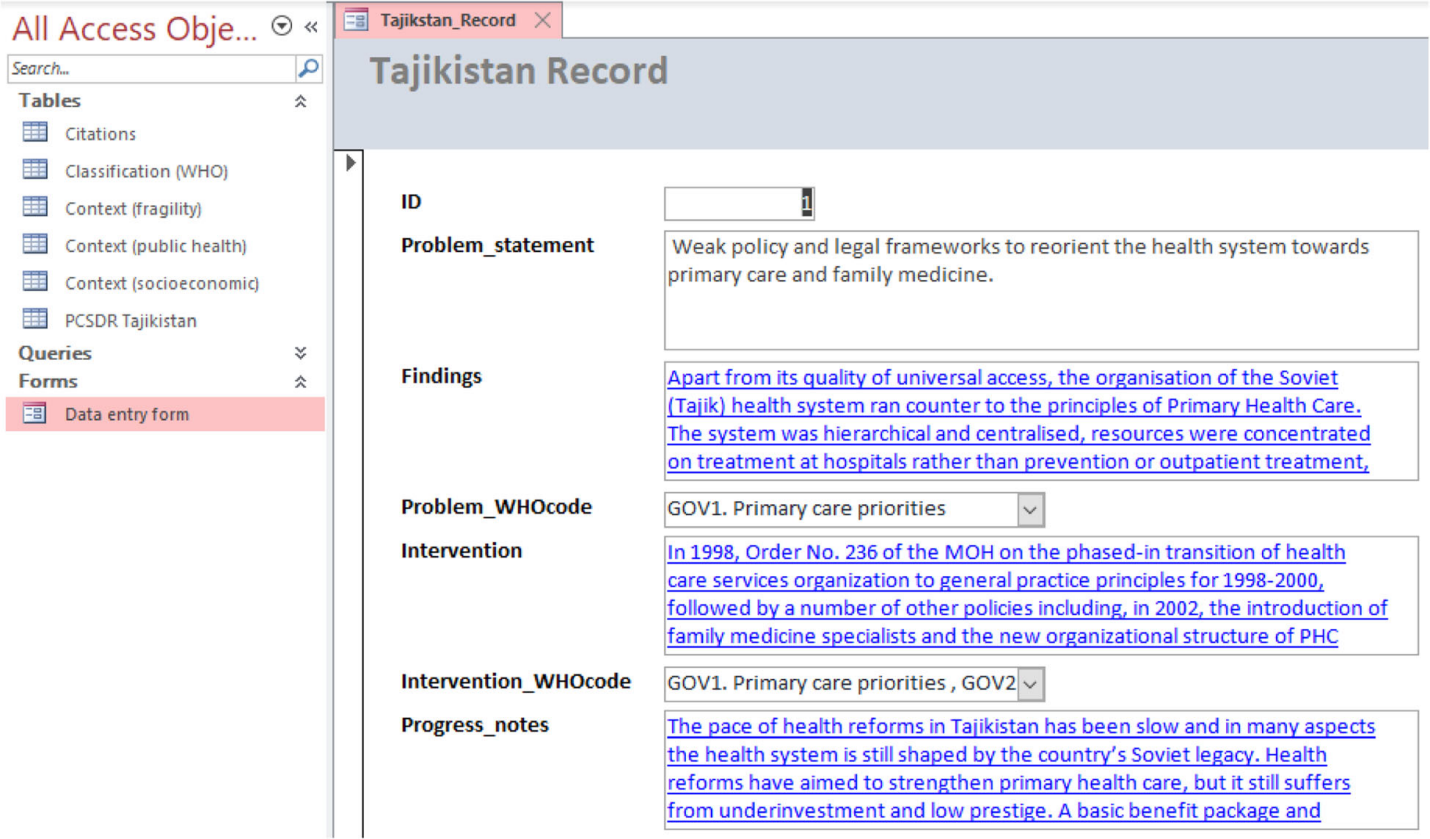
PCSDR Data entry form (screenshot)

The PCSDR data entry form is to be used for data entry. Each problem statement is assigned a unique ID, which is critical for maintenance of data and referential integrity, as well as for enabling the relational database’s key functionalities such as queries (Fig. 5).

Where possible, problem statements should be structured for the clear identification of their respective problem system and performance attribute(s) (i.e. problem statement = problem system component [structure, process or outcome] + attribute). The data entry form’s multi-value fields enable multiple codes to be recorded for respective problem and intervention systems.

The data entry form allows entry of hyperlinked free text in the “Findings”, “Intervention” and “Progress notes” fields. Such content enables the contextualization of information within the PCSDR and informs the further development of classification content. A bibliography of sources cited is available for download from the ‘Citations’ table.

### The PCSDR table

**Fig. 6:**
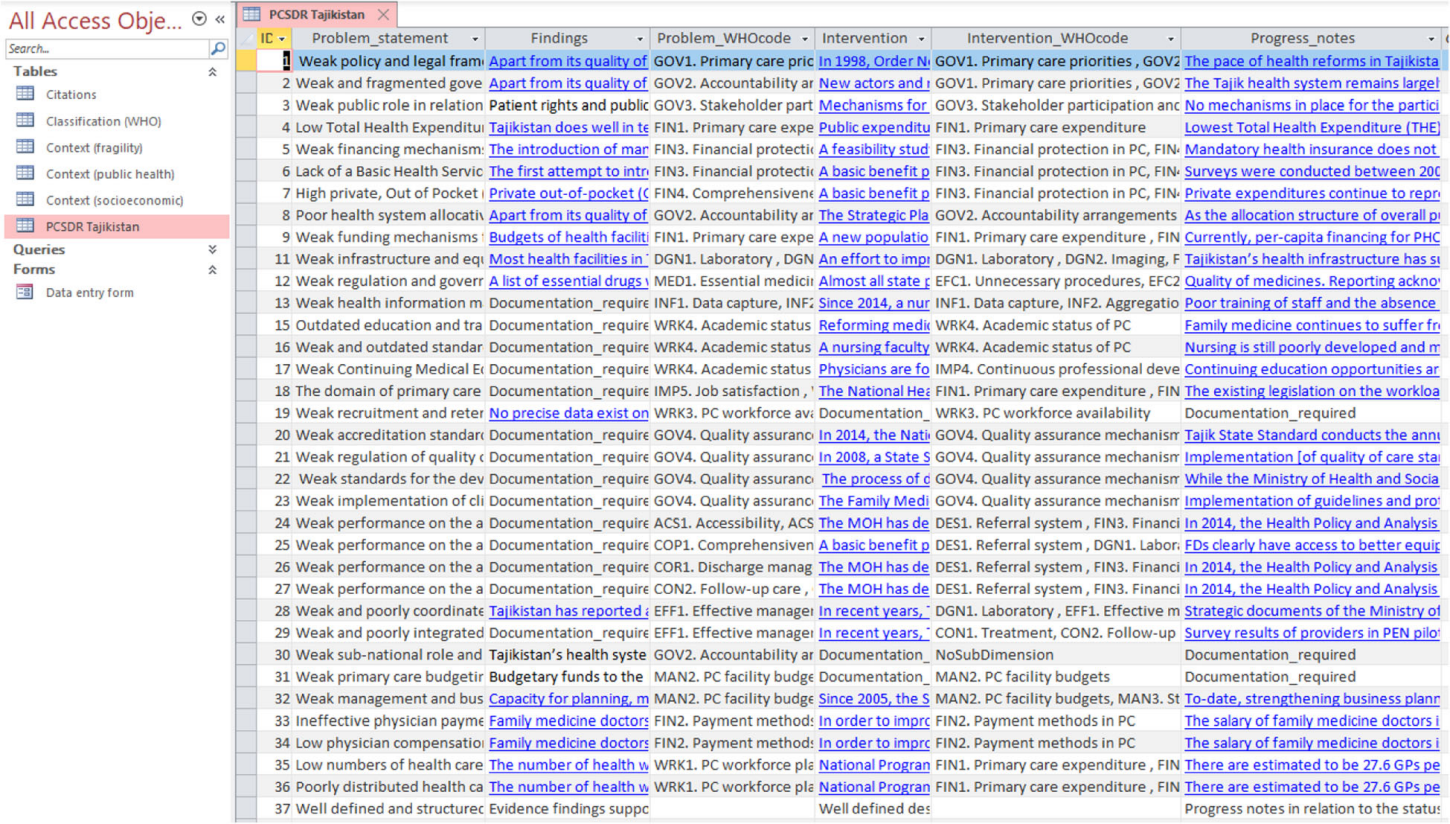
Tajikistan PCSDR table (screenshot)

The totality of entered data is structured and stored in the PCSDR table referred to as the “Record” table (Fig. 6). The contents of Tajikistan’s PCSDR were also exported to MS Word (Additional File 2).

Tajikistan’s completed PCSDR is comprised of 36 problem statements. It conveys the narrative of a middle-income country in transition, attempting to orient its health care system towards the principles of universal health coverage and a primary health care approach. The content of the problem statement list is mainly comprised of foundational and structural domains, such as governance, policy, financing, funding, infrastructure, human resources and education, depicting Tajikistan’s efforts related to redressing the structural legacy of its inherited Soviet system (Table 1).

**Table 1:** Snapshot of a query of Tajikistan’s Problem Statement list and Problem System (Probα) codes (the full list generated by the query is available in the Supplementary file)

ID	Problem_statement	Problem_WHOcode (Probα Code)
1	Weak policy and legal frameworks to reorient the health system towards primary care and family medicine.	GOV1. Primary care priorities
2	Weak and fragmented governance and accountability mechanisms related to the development and strengthening of primary care.	GOV2. Accountability arrangements
3	Weak public role in relation to the governance and/or organization of the primary care system.	GOV3. Stakeholder participation and engagement
4	Low Total Health Expenditure (THE)	FIN1. Primary care expenditure
5	Weak financing mechanisms – revenue collection, pooling, and coverage.	FIN3. Financial protection in PC, FIN4. Comprehensiveness of financial protection for PC services
6	Lack of a Basic Health Services Package (BHSP) / Basic Benefits Package.	FIN3. Financial protection in PC
7	High private, Out of Pocket (OOP) and informal expenditure, as a percent of Total Health Expenditure (THE).	FIN4. Comprehensiveness of financial protection for PC services
8	Poor health system allocative efficiency.	GOV2. Accountability arrangements
9	Weak funding mechanisms for primary care facilities.	FIN1. Primary care expenditure
11	Weak infrastructure and equipment.	DGN1. Laboratory, DGN2. Imaging, STR 1. Basic amenities, TCH1. Basic technology

Similar to other post-Soviet countries undergoing structural adjustment-type reforms, primary care system development in Tajikistan was not generally done using a problem-oriented approach [28, 29]. Therefore, interventions were not often underpinned by clearly defined problems or evidence. This is reflected by the limited content of the PCSDR’s “Findings” field. Multi-value fields for problem systems and intervention systems may also indicate that Tajikistan’s performance bottlenecks are potentially not well defined. It may also reflect the inherent challenge of long-term and large-scale reforms, spanning varied policy cycles and leadership. In the absence of well-developed systems for recording and sharing information, the exchange of information on prior decisions taken often lack this feedback. Furthermore, this may also reflect limitations in the classification system applied.

### Investigating complexity: querying relationships between PCSDR fields

Multi-value fields for intervention systems may also indicate that problems were appropriately addressed by targeting multiple underlying causal system areas. Elucidating the complex network relationships between Problem (Probα) and Intervention Systems (Txα) enables analysts and researchers to understand the interactions and dynamics within the health care system, which in turn enables more effective and efficient development activities. The MS Access relational database query functionality enables interrogation of these relationships. For example, an analyst can easily query what Intervention Systems (Txα) relate to specific Problem Systems (Probα), and vice versa.

**Fig. 7:**
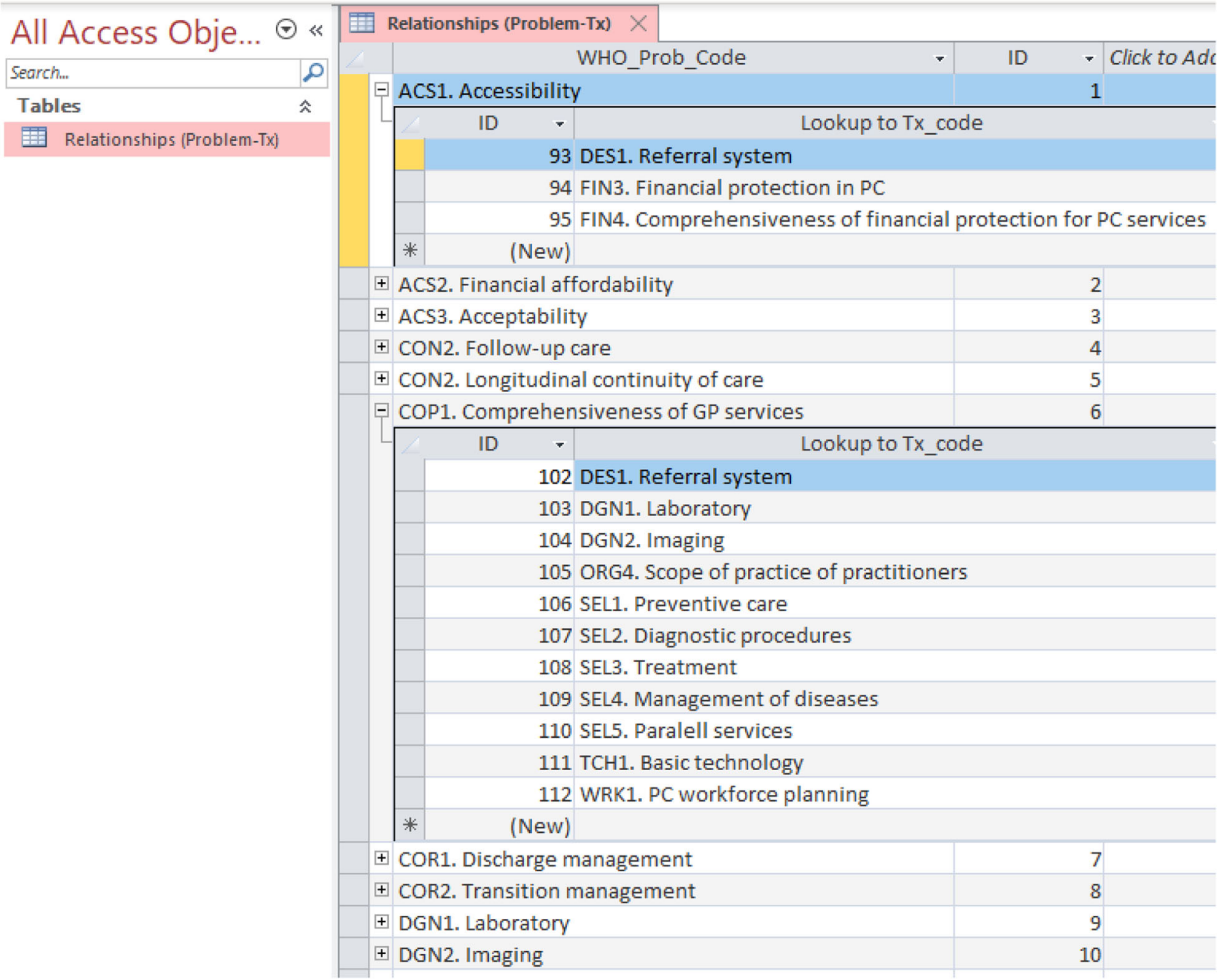
Relationships between Problem System (Probα) Codes and Intervention System (Txα) Codes (screenshot)

For example, Fig. 7 shows how “Accessibility” (ACS1) and “Comprehensiveness of GP Services” (COP1) Problem System (Probα) domains are addressed via a number of different Intervention Systems (Txα). See ‘Additional Files 3 and 4’ for full access to the query result tables.

### Contextual tables

**Fig. 8:**
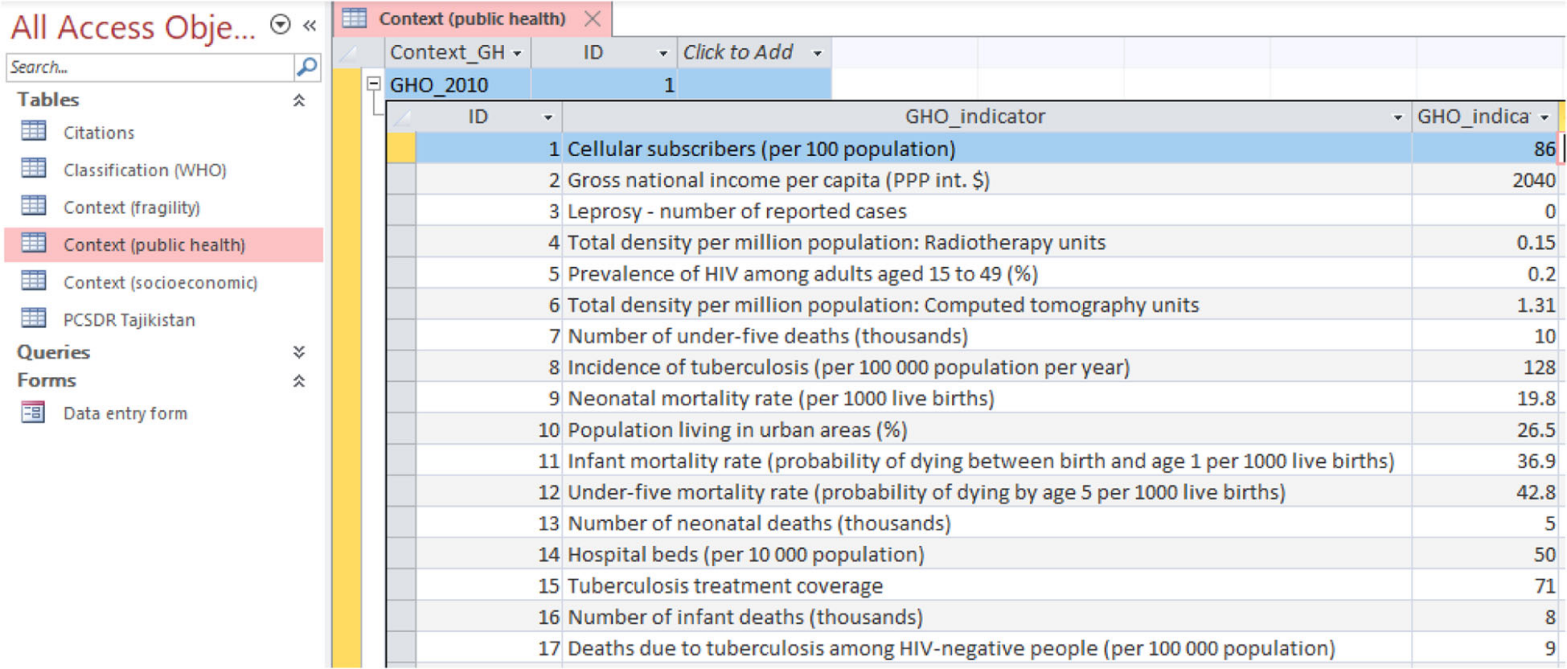
Public health context table comprised of WHO Global Health Observatory (GHO) indicators, by year (screenshot)

Contextual tables providing indicators on Tajikistan’s state fragility, public health, and socioeconomic contextual indicators are organized by database type and year, to facilitate ease of use. Figure 8 provides a snapshot of a Public health context table comprised of WHO Global Health Observatory (GHO) indicators, by year (screenshot).

By including these contextual tables in the relational database, their respective content could hypothetically be linked with the PCSDR if fields for time intervals are included. This could allow for useful research that integrates the work of different fields (e.g. primary care system development and state fragility). Snapshots of the fragility and socioeconomic contextual tables can be viewed in Additional file 5 (Fig. 9 and 10).

## Discussion

Systems-oriented evidence-based decision making is contingent on the application of scientific knowledge to problems and issues of concern within particular contexts [6]. For this to occur, knowledge must be systematically structured in a way that is rational, conceptually sound and valid [16, 17, 30]. The structuring, application and development of knowledge require robust data input and management systems [3].

The PCSDR presents an example of a basic data source system via a structured record for input and classification of different data types from myriad sources, as well as a data management system that enables the structuring, storing and processing of data. The structured data entry presents an opportunity for primary care system analytics, modeling and reporting functions, which in turn can directly support research, evaluation and evidence-informed decision-making.

To enable its practical and effective use in the context of real-world complex and dynamic primary care systems, the PCSDR was designed be conceptually sound (i.e. problem and systems-oriented), content valid (i.e. operationalize scientific classification systems), and fit-for-purpose (i.e. enable evidence-based primary care system development functions).

### Problem-orientation

Lasswell’s concept of problem-orientation has recently been recognized as being fundamental in relation to building state capacity and international development functions [18, 19, 28]. For example, the Problem-Driven Iterative Adaptation (PDIA) approach now underpins the Building State Capability programme at Harvard University’s Center for International Development, and is being leveraged by various World Bank country programmes [28, 31]. Similar to other post-Soviet countries undergoing structural adjustment-type reforms, primary care system development in Tajikistan was primarily driven by a focus on system inputs rather than a problem-oriented approach able to unpack the root cause of suboptimal performance and services delivery bottlenecks [29]. In effect, interventions were often not underpinned and tailored to clearly defined problems nor decisions based on the fluid exchange and use of evidence overtime, across policy cycles and periods of system leadership.

The PCSDR is a tool that can systematically enable a problem-oriented approach for primary care system development. Its ability to both create a system of recording and meaningfully associate data is particularly relevant for countries undergoing long-term and large-scale transitions.

### Systems-orientation

The PCSDR enables the use of systems-oriented primary care frameworks and classification systems for primary care system development activities (in the case of this study, the PHC-IMPACT framework and classification) [12, 14, 32]. The PCSDR enables users to perform the following functions:

- data collection and entry for different types of data from myriad sources;
- coding of PCSDR data using scientific classification systems (e.g. PHC-IMPACT framework classification);
- measurement of primary care system performance;
- assessment of determinants of performance;
- measurement and assessment of changes in performance states over time (through longitudinal use of the PCSDR);
- assessment of system interactions and interventions associated with changes in performance; and
- access to external databases via data linkage (e.g. relevant contextual datasets).

The PCSDR’s simple structure enables these functions to be conducted in a logical, scientific and systematic manner. Its systems-oriented classification systems and taxonomies of problem-oriented development functions (i.e. F(x) fields] promote scientific progress by enabling research, analysis and evaluation.

The scientific development of primary care system classifications and the practical use of the PCSDR are complementary and codependent. Conceptually-sound and granular classification systems enable effective operationalization of the PCSDR, which in turn, generates, structures and stores content knowledge, enabling further refinement and development of classification systems.

### Data management system: structuring knowledge to enable analytics

A relational database enables data to be structured and stored in the form of a semantic network [27]. This approach to structuring knowledge operationalizes a systems paradigm, whereby knowledge is conceived as a complex network of relationships [16, 17]. The relational database provides a framework that captures statements regarding the content and dynamics of relationships between the PCSDR’s fields. Knowledge is thereby contextualized, rendering its application to specific problems within particular settings valid and effective [17, 30]. Such a data management approach enables a contextualized approach to evidence-based decision making [3, 5].

By structuring data, the relational database enables analysts and researchers to conduct queries for descriptive and analytical functions [3, 5]. Queries of fields, keywords and/or classification codes (and combinations thereof) enable explorative and descriptive functions related to the investigation, validation (i.e. completeness and accuracy) and development of the PCSDR’s content. This study highlighted how an analyst can easily query content related to Tajikistan’s problem list (e.g. Problem Statements and Problem System codes).

Queries of relationships between the PCSDR’s fields (e.g. between Problem [Probα] and Intervention System [Txα] codes) are critical from a scientific research and knowledge perspective, since they enable key health services research and health system performance assessment functions. For example, an understanding of the complex influence of interventions on system interactions would provide useful insights related to their respective effectiveness and efficiency. Tajikistan’s PCSDR provided a clear example of how problems are often addressed by targeting multiple underlying causal system areas, which in turn often influence other problem areas. The PCSDR enables this systems complexity to be systematically structured and interrogated.

This study also demonstrated how the relational database enables strategic linkage with external datasets containing relevant and useful contextual data, such as demographic, epidemiological and socioeconomic, political indicators. Such simple functionalities are not only efficient and practical for analysts; they can enable effective cross-disciplinary research through data linkage between the respective databases [33–35].

### Future development

The PCSDR provides a potentially conceptually sound, effective and practical approach that can contribute towards enabling a data-driven approach for primary care system development. For example, the PCSDR can potentially be adapted and leveraged to enable operationalization of the mandate of the newly established WHO ‘Division of Data, Analytics and Delivery’ relating to “enhancing the collection, storage, analysis and usage of data to drive policy change in countries” [2]. The tool could also be useful for research networks, think tanks and expert groups at the EU level (such as the EU Expert Group on Health Systems Performance Assessment and the European Forum for Primary Care), by enabling primary care system policy makers and practitioners to systematically share best practices and experiences. The tool may also be interesting and useful for the scientific community, as health policy scientists could better monitor and assess the impact of healthy policies and system changes if they were supported by the availability of such a tool.

The PCSDR can enable and systematize important policy and planning governance functions by supporting Ministries of health through a consolidated and structured database. Updates and changes to the underpinning evidence base are recorded, thereby enabling transparency. As earlier noted, the record facilitates institutional memory that is often lost between policy cycles. The availability of a structured and consolidated database is especially relevant in contexts where there are many international stakeholders and financiers, such as in the case of Tajikistan where organizations operate in siloes and have their own data structures and reporting mechanisms.

At an international level, PCSDR datasets comprised of longitudinal data across countries has the potential to support health services research to leverage statistical and machine learning tools in order to identify complex patterns, and to accurately understand the structure and function of primary care systems. This would enable cross-learning and the development of decision support tools that yield contextualized knowledge on the influences of interventions to primary care system performance. Such assessments of the effectiveness and efficiency of interventions foster quality improvement cycles and innovative system design work. Together, the PCSDR, classification systems and structured relational databases may form the fundamental building blocks of future learning primary care systems.

Embarking upon this ambitious future vision requires an awareness of the current realities of the field of international primary care system development. The PCSDR prototype presented in this paper was purposefully designed to align with and reflect the current state of the field, which remains in its infancy in relation to defining, standardizing and classifying its core content (i.e. functions and subject matter) [7, 12].

The PCSDR presented in this paper has many technical and operational limitations, as it is a preliminary prototype that aims to demonstrate basic proof-of-concept. Ultimately, health services researchers and development experts must decide on collaborative processes to properly develop its content and design. For example, some key areas for further reflection and consideration may include:

- What fields should comprise the core content of the PCSDR?
- How should the status and content of the problem list be designated (e.g. active and inactive problems) and updated (e.g. redefining and reclassifying problems)?
- What are the rules for data entry?
- What classification system(s) should be used?
- How should date and time stamps be incorporated?
- How should the ‘Progress Notes’ field be updated? (i.e. analogous functionality to ‘Encounter Notes’ in POMRs)
- How should citations be integrated and linked?
- What contextual databases are relevant and useful for inclusion, what are the optimal mechanisms for their linkage?
- What types of decision support tools should be developed and integrated (e.g. a tool that flags potential problem system areas via identification of “weak” performance indicators in linked databases; or linkage of the PCSDR with the Primary Health Care Performance Initiative (PHCPI) Evidence Tool Database).
- What type of database management system should be used?
- What standard operating procedures and protocols need to be developed to enable proper operationalization and testing?

Furthermore, the development of practical evaluation frameworks that enable the testing of the type of outputs produced by the PCSDR under different scenarios is required. Such studies would provide insights into the utility and effectiveness of the PCSDR, particularly in relation to alternative approaches and the status quo, and to investigate what inputs, resources and training are required to enable the efficient and effective use of the tool. It is also essential to explore whether and how the PCSDR could potentially be integrated with other planning tools currently in use.

In addition to the PCSDR’s conceptual and technical development, scientific and organizational consensus is required in relation to systematically adopting problem-orientation for primary care system development [18, 19, 28, 31]. Furthermore, early users of the PCSDR will have to contend with the difficulties of having to code with rather rudimentary classification systems, as was the case in this study. The PHC-IMPACT framework has recently been developed and is expected to continue to be refined and improved upon through its use over time. These are all similar issues that continue to face other fields such as clinical medicine, though through continued use and development, classification systems for clinical medicine have greatly improved over time which is promising for proponents of the overall approach espoused by the PCSDR [36].

## Conclusions

Certain attributes of systems development functions are essential and universal, namely: problem-orientation, systems-orientation and contextuality [18]. These are undoubtedly desirable attributes for the field of primary care system development. This study demonstrates that these attributes can be operationalized systematically and consistently through the rigorous use of a structured data systems approach, as shown by the PCSDR.

The PCSDR structures data in a way that enables the development of measurable scientific knowledge related to the basic anatomy (structure) and physiology (function) of primary care systems. Such knowledge enables research pertaining to the aetiology of system dysfunction which, in turn, enables analysts and researchers to devise and test mechanisms and interventions related to influencing and rectifying dysfunction.

The PCSDR enables the structuring of all such functions, which enables analysts to meaningfully assess the value (i.e. effectiveness and efficiency) of respective interventions. The PCSDR thereby enables the field of primary care system development to transform into a professional and scientific domain, amenable to assessment and improvement.

## Supplementary information

**Additional file 1.** PCSDR Database for Tajikistan. The database is comprised of the PCSDR table, the PCSDR data entry form, the WHO PHC-IMPACT classification system, citations and contextual tables.

**Additional file 2.** Tajikistan PCSDR table.

**Additional file 3.** Query of relationships between Txα and Probα codes for Tajikistan’s PCSDR.

**Additional file 4.** Query of relationships between Probα and Txα codes for Tajikistan’s PCSDR.

**Additional file 5.** Supplementary file containing the following: the full Table 1, Fig. 9 and Fig. 10. Table 1. Query of Tajikistan’s Problem Statement list and Problem System (Probα) codes. Fig. 9. Socioeconomic context table comprised of UNData indicators, by year (screenshot). Fig. 10. Fragility context table comprised of Deutsches Institut für Entwicklungspolitik (DIE) Constellations of State Fragility and Fragile State Index (FSI) indicators, by year (screenshot).

## Data Availability

All data generated or analysed during this study are included in this published article (and its Additional Files).

## Abbreviations

PCSDR: Primary Care System Development Record
WHO: World Health Organization
PCET: Primary Care Evaluation Tool
PHC-IMPACT: European Tool for Monitoring Impact, Performance and Capacity of Primary Health Care
F(x): Primary care system development functions
POMR: Problem-Oriented Medical Record
∆: Time
α: System
Probα: Problem System
Txα: Intervention System
FSI: Fragile State Index
DIE: Deutsches Institut für Entwicklungspolitik
PDIA: Problem-Driven Iterative Adaptation
PHCPI: Primary Health Care Performance Initiative
