# Supplementary material for: A problem-oriented systems approach to primary care system development: development and initial testing of the problem-oriented primary care system development record
Source: BMC Health Serv Res. 2020 Aug 1;20:706. doi: 10.1186/s12913-020-05581-z (PMC7395390; doi:10.1186/s12913-020-05581-z)
Supplement: Supplementary file 2 — Additional file 2. Tajikistan PCSDR table. [file 12913_2020_5581_MOESM2_ESM.rtf]

ID	Problem_statement	Findings	Problem_WHOcode	Intervention	Intervention_WHOcode	Progress_notes	
1	 Weak policy and legal frameworks to reorient the health system towards primary care and family medicine.	Apart from its quality of universal access, the organisation of the Soviet (Tajik) health system ran counter to the principles of Primary Health Care. The system was hierarchical and centralised, resources were concentrated on treatment at hospitals rather than prevention or outpatient treatment, and the workforce was highly specialised, rather than making effective use of generalist physicians and nurses ) (Parfitt and Cornish 2007)	GOV1. Primary care priorities 	In 1998, Order No. 236 of the MOH on the phased-in transition of health care services organization to general practice principles for 1998-2000, followed by a number of other policies including, in 2002, the introduction of family medicine specialists and the new organizational structure of PHC facilities. (“Review of the National Programme on the Development of Family Medicine 2011-2015 in Tajikistan” 2016)
 
In March 2002, the Government of Tajikistan approved a health reform programme, to reallocate resources from hospitals to PHC (Parfitt and Cornish 2007)
 
The National Health Strategy of Tajikistan 2010-2020 highlighted the development of PHC based on family medicine practice among the main health reform priorities during this period. That same year, the law on family medicine defining regulatory functions, as well as competencies, rights and obligations of family medicine specialists was enacted (Order No. 676, 29 December 2010). The law triggered the development and further approval of the National Programme on Family Medicine development in Tajikistan for 2011-2015. (“Review of the National Programme on the Development of Family Medicine 2011-2015 in Tajikistan” 2016)
 
New actors and responsibilities at the national level and advancements in policy have secured impressive gains in institutional capacity within the health system. Efforts to establish family medicine practice have included formalizing a legal framework for family medicine and numerous policies approving the organizational structure of primary care facilities and introduction of family medicine specialists. Moreover, there are a number of new macro-level family medicine actors within the health system, including the Republican Centre for Family Medicine responsible for implementation of family medicine policies. There are also new actors working across levels of the health system, such as the State Supervision Service of Medical Activities (Khadamot) and the Evidence-based Medical Centre (EBMC). This institutional capacity has built a critical mass of technical resources for health services delivery nationally. (“Review of the National Programme on the Development of Family Medicine 2011-2015 in Tajikistan” 2016)
 
In 2010, the MOH established the Inter-sector Coordination Board for Family Medicine for the continued strengthening of PHC, including family medicine. The deputy minister of health is chairman of this board. The board itself has representation from the MOH, local institutions and Development Partners. The purpose of this council is to promote political dialogue to further strengthen the primary care system in the country (Order No. 416, 7 December 2013). (“Review of the National Programme on the Development of Family Medicine 2011-2015 in Tajikistan” 2016)	GOV1. Primary care priorities , GOV2. Accountability arrangements, GOV3. Stakeholder participation and engagement	The pace of health reforms in Tajikistan has been slow and in many aspects the health system is still shaped by the country's Soviet legacy. Health reforms have aimed to strengthen primary health care, but it still suffers from underinvestment and low prestige. A basic benefit package and capitation-based financing of primary health care have been introduced as pilots but have not yet been rolled out to the rest of the country. The National Health Strategy envisages substantial reforms in health financing, including nationwide introduction of capitation-based payments for primary health care and more than doubling public expenditure on health by 2020; it remains to be seen whether this will be achieved  (Khodjamurodov et al. 2016)
 
If the main aim of the health reforms is to improve the performance of the health system in offering affordable and equitable health care services, the financial barriers that patients face when utilising PHC must be addressed. In fact, the BBP policy alone proves not to be a sufficient instrument if not accompanied by parallel efforts to improve delivery of PHC services such as incentives for family doctors to deliver quality care, improved prescription rationality, and measures to guarantee an affordable and equitable drug supply system. Such measures require increased public investments in the health sector, as seen in neighbouring countries.  (Schwarz et al. 2013)
 
Specific stimulation was achieved through the development of the national programme on family medicine for 2011–2015 and its related plan. A recent report from the programme states that 10 outpatient facilities and 73 family medicine units are currently operational in the country, with 546 family medicine specialists and 655 family health nurses working at primary care level(1). Despite several strategic policy documents and regulation favouring primary care, the pace of progress towards family medicine is nevertheless slow.  (“EVALUATION OF THE STRUCTURE AND PROVISION OF PRIMARY CARE IN TAJIKISTAN” 2014)
 
In 2015, the Family Medicine Programme came to a close. A WHO review has highlighted opportunities for continued improvement. The guiding principles of the WHO European Region health policy framework, Health 2020 (WHO Regional Office for Europe 2013), and WHO global framework for integrated and people centred services delivery (WHO 2015b), continue to put forth this vision in order to truly accelerate improvements in population and individual health outcomes. (“Review of the National Programme on the Development of Family Medicine 2011-2015 in Tajikistan” 2016)
 
Despite a prosperous economic period since 2000 and increasing international aid investments, the lack of political stewardship has led to weak engagements in health reforms that have so far not shown to be sufficient instruments to address households financial burden in accessing health care. Thus, though the proportion of OPE in the total health expenditure may have declined in the last years as an effect of increased government and foreign investments, this may not translate into a reduction of direct payments from patients in absolute figures. An internal evaluation of the Ministry of Health on the impact of the BBP conducted in 2008 at the hospital level comparing trends before and after the introduction of BBP in pilot and control districts has shown no reduction in the overall patient financial burden with utilising services, with the exception of deliveries and exempt patients  .. (Schwarz et al. 2013)
 
Informal payments are not apprehended in this paper as a problem of corrupt practices, but as a symptom of the general condition of the Tajik health system that is under-financed and that lacks efficient and strong policy instruments. The findings of this analysis show that the overall expenditures of primary health care patients in four study districts in Tajikistan, adjusted for inflation, have doubled between 2005 and 2011. This observation is mainly explained by an increase in the expenditures on medicine. In fact, not only do patients spend more money on prescribed drugs, but they are being prescribed and they obtain their medicine in larger proportions. (Schwarz et al. 2013)	
2	Weak and fragmented governance and accountability mechanisms related to the development and strengthening of primary care.	Apart from its quality of universal access, the organisation of the Soviet (Tajik) health system ran counter to the principles of Primary Health Care. The system was hierarchical and centralised, resources were concentrated on treatment at hospitals rather than prevention or outpatient treatment, and the workforce was highly specialised, rather than making effective use of generalist physicians and nurses .. (Parfitt and Cornish 2007)	GOV2. Accountability arrangements	New actors and responsibilities at the national level and advancements in policy have secured impressive gains in institutional capacity within the health system. Efforts to establish family medicine practice have included formalizing a legal framework for family medicine and numerous policies approving the organizational structure of primary care facilities and introduction of family medicine specialists. Moreover, there are a number of new macro-level family medicine actors within the health system, including the Republican Centre for Family Medicine responsible for implementation of family medicine policies. There are also new actors working across levels of the health system, such as the State Supervision Service of Medical Activities and the Evidence-based Medical Centre (EBMC). This institutional capacity has built a critical mass of technical resources for health services delivery nationally. .. (“Review of the National Programme on the Development of Family Medicine 2011-2015 in Tajikistan” 2016)
 
In 2010, the MOH established the Inter-sector Coordination Board for Family Medicine for the continued strengthening of PHC, including family medicine. The deputy minister of health is chairman of this board. The board itself has representation from the MOH, local institutions and Development Partners. The purpose of this council is to promote political dialogue to further strengthen the primary care system in the country (Order No. 416, 7 December 2013). (“Review of the National Programme on the Development of Family Medicine 2011-2015 in Tajikistan” 2016)	GOV1. Primary care priorities , GOV2. Accountability arrangements, GOV3. Stakeholder participation and engagement, GOV4. Quality assurance mechanisms, MAN3. Strategic planning 	The Tajik health system remains largely state owned and administered and the structure is generally (though not universally) hierarchical. However, the coordination between the national level, the oblast and rayon administrations and local health facilities is compromised by unclear accountability arrangements (such as those arising from the abolition of rayon-level health departments in 2012) and the absence of a formal mandate and authority for managing localized services. While Tajikistan is still heavily centralized in terms of health policy and strategy, it is a fiscally decentralized system, including in health financing. .. (Khodjamurodov et al. 2016)
 
 
Institutional harmonization for clear lines of accountability between the national level and regional and local facilities is required. Following the abolishment of rayon-level health departments in 2012, there is a critical need at present for a subnational coordinating mechanism and clarified roles and responsibilities. While serving as a means to promote policy dialogue and coordination, The PHC Coordination Council has not alleviated the need to redefine lines of accountability between levels of the health system. .. (“Review of the National Programme on the Development of Family Medicine 2011-2015 in Tajikistan” 2016)
 
Attention should be paid to possible inequities arsing as a consequence of decentralization. Growing regional autonomy may result in differences in health care service funding between districts and regions, which may have implications for (geographic) inequities. .. (“EVALUATION OF THE STRUCTURE AND PROVISION OF PRIMARY CARE IN TAJIKISTAN” 2014) 
 
Despite a prosperous economic period since 2000 and increasing international aid investments, the lack of political stewardship has led to weak engagements in health reforms that have so far not shown to be sufficient instruments to address households financial burden in accessing health care. Thus, though the proportion of OPE in the total health expenditure may have declined in the last years as an effect of increased government and foreign investments, this may not translate into a reduction of direct payments from patients in absolute figures. An internal evaluation of the Ministry of Health on the impact of the BBP conducted in 2008 at the hospital level comparing trends before and after the introduction of BBP in pilot and control districts has shown no reduction in the overall patient financial burden with utilising services, with the exception of deliveries and exempt patients  .. (Schwarz et al. 2013)	
3	Weak public role in relation to the governance and/or organization of the primary care system.	Patient rights and public involvement in health policy are still in their infancy. (Khodjamurodov et al. 2016)	GOV3. Stakeholder participation and engagement	Mechanisms for involving communities in the organization of the local health system that are being tested in some pilots, but have not yet been established at national level.  (Khodjamurodov et al. 2016)	GOV3. Stakeholder participation and engagement, GOV4. Quality assurance mechanisms	No mechanisms in place for the participation of patients or the general public in the policy-making process. Surveys on patient satisfaction have only been carried out occasionally and point in contradictory directions. . (Khodjamurodov et al. 2016)
 
Patient rights are set out in the Law on Public Health Protection, with several other laws having indirect implications for patient protection. The 2002 health reform programme addressed patient information and involvement in service planning and delivery. To date, no formal organization or association for defending patient rights or informing the public about health care issues exists. Patient complaint procedures are in place and health facilities are obliged to provide a complaints box for patients to submit comments, which are then addressed by committees or at higher administrative level, including the Ministry of Health. (“EVALUATION OF THE STRUCTURE AND PROVISION OF PRIMARY CARE IN TAJIKISTAN” 2014)	
4	Low Total Health Expenditure (THE)	Tajikistan does well in terms of total health expenditure as a percentage of GDP (considering its socioeconomic position as the poorest country in the region), but its health expenditure in absolute terms is by far the lowest in the region and the share of public sector expenditure is among the lowest. . (Khodjamurodov et al. 2016)	FIN1. Primary care expenditure 	Public expenditure as a share of total health expenditure has been growing slightly in recent years. While it remains low in absolute and relative terms, the structure shows a favourable trend towards increased spending in PHC. . (“Review of the National Programme on the Development of Family Medicine 2011-2015 in Tajikistan” 2016)	FIN1. Primary care expenditure 	Lowest Total Health Expenditure (THE) per capita in the WHO European Region, US$ 170 (PPP) in 2013. . (Khodjamurodov et al. 2016)
 
One of the challenges will be to direct sufficient public resources into the health system. The National Health Strategy of the Republic of Tajikistan 2010–2020 envisages that public expenditure on health as a share of GDP should reach 3.4% in 2015 and 4.4% in 2020 (Ministry of Health and Social Protection, 2014). In 2013, it accounted for 2.1% (“Review of the National Programme on the Development of Family Medicine 2011-2015 in Tajikistan” 2016)
 
Private expenditures continue to represent the highest share of total health expenditure; in 2012, out-of-pocket expenditures accounted for 62.5% of total health expenditures (MOH 2014a) (“Review of the National Programme on the Development of Family Medicine 2011-2015 in Tajikistan” 2016)	
5	Weak financing mechanisms – revenue collection, pooling, and coverage .	The introduction of mandatory health insurance has been envisaged for many years but was postponed several times. Voluntary health insurance is virtually non-existent. . (Khodjamurodov et al. 2016)
  
Pooling mechanisms are still underdeveloped and there is no real mechanism for purchasing health services. . (Khodjamurodov et al. 2016)
 
Private out-of-pocket (OOP) payments as a percentage of total health expenditure was one of the highest in the WHO European Region, reaching 60.1% . (Khodjamurodov et al. 2016)
 
Most government revenue is generated locally and oblasts determine to a large degree the formation of local health budgets from which funds are allocated to the health facilities. While Tajikistan is still heavily centralized in terms of health policy and strategy, it is a fiscally decentralized system, including in health financing. . (Khodjamurodov et al. 2016)	FIN3. Financial protection in PC, FIN4. Comprehensiveness of financial protection for PC services	A feasibility study on the introduction of mandatory health insurance was conducted in March 2013 (O'Dougherty et al., 2013). The study identified a number of preconditions for the implementation of mandatory health insurance, including the availability of institutional structures, functions and a precise definition of mutual relations between the key interested ministries and agencies (HPAU, 2013c; O'Dougherty, Zues & Akkazieva, 2014). One of the challenges is that the Ministry of Finance does not support the introduction of mandatory health insurance because of a lack of fiscal space and lacking capacity within the health system. A decision was made to postpone the  Introduction of mandatory health insurance until 2017, while the country tries to put in place the preconditions set out in the feasibility study (O'Dougherty, Zues & Akkazieva, 2014). (Khodjamurodov et al. 2016)	FIN3. Financial protection in PC, FIN4. Comprehensiveness of financial protection for PC services	Mandatory health insurance does not exist, but its introduction has been envisaged for several years. . (Khodjamurodov et al. 2016)
 
The introduction of mandatory health insurance has been envisaged for many years but was postponed several times. Voluntary health insurance is virtually non-existent. . (Khodjamurodov et al. 2016)
 
According to National Health Accounts data, private health insurance was estimated to account for only 0.1% of total health expenditure in 2012 (Khodjamurodov et al. 2016)
 
General government expenditure largely relies on the resources of oblast and rayon authorities, which contributed 81.2% of government expenditure in 2012. As this makes health financing dependent on local resources, it compounds regional inequalities, with the poorest regions spending the least on health per person. . (Khodjamurodov et al. 2016)
 
There are pronounced inequities in health care resources across oblasts and rayons, and the distribution of public spending tends to be inequitable as well, benefiting the rich more than the poor . (Khodjamurodov et al. 2016)
 
Pooling mechanisms are still underdeveloped and there is no real mechanism for purchasing health services. . (Khodjamurodov et al. 2016)
 
Private out-of-pocket (OOP) payments as a percentage of total health expenditure was one of the highest in the WHO European Region, reaching 60.1% . (Khodjamurodov et al. 2016)
 
Per capita health expenditure varies across oblasts and is not related to social or health needs, with the poorest oblasts spending the least per capita. The Ministry of Health and Social Protection has long held the view that pooling of funds at least at the oblast level is a precondition for health financing reform and critical to increasing equity and financial risk protection. However, to date, the Ministry of Health and Social Protection and the Ministry of Finance have not reached consensus on the pooling of funds (Ministry of Health and Social Protection, 2014; O'Dougherty, Zues & Akkazieva, 2014). (Khodjamurodov et al. 2016)
 
The BHSP reforms do not include an accompanying institutional reform for the pooling of resources necessary to address the existent financial unbalances across health care levels and across districts due to a fragmented budget formulation .  (Schwarz et al. 2013)	
6	Lack of a Basic Health Services Package (BHSP) / Basic Benefits Package.	The first attempt to introduce the guaranteed benefit package in 2005 had several shortcomings in design and implementation (Saifuddinov, Severoni & Artykova, 2009) and its implementation led to considerable public dissatisfaction; it was suspended after only two months, in October 2005. (Khodjamurodov et al. 2016)	FIN3. Financial protection in PC	A basic benefit package was adopted in principle in 2007, with the aim of defining which services should be provided at no cost (focused on essential primary and emergency care) and formalizing additional payments for others (as opposed to current informal payments). However, it is still in pilot mode and has so far only been extended to 14 of the country's 65 districts. .  (Khodjamurodov et al. 2016)
 
The basic benefits package is covered through the state budget and mainly comprises basic medical services provided by primary health care facilities. . (Khodjamurodov et al. 2016)
 
The BBP aims at strengthening the role of family doctors as gate keepers of the health system by setting a lower co-payment fee at the secondary level for patients who have been formally referred by their family doctor . (Schwarz et al. 2013)
 
A constitutional amendment removing the right to free health care was approved by a national referendum in June 2003, allowing the government to introduce co-payments for all state-run health services. This marked an important break with the past and indicated the commitment of the government to implement reforms in the health sector. The constitutional amendment allowed the government to prioritize the allocation of health resources in line with the state-guaranteed essential health services and to introduce co-payments for other health services. . (Khodjamurodov et al. 2016)
 
In 2013, the HPAU carried out a feasibility study to explore potential expansion of the basic benefit package to six additional rayons. The key findings of this study highlighted that existing budgetary allocations would be insufficient to cover the costs of the programme, but despite this conclusion the MOH scaled up the BBP these rayons within the standard budget provided by the Ministry of Finance (MOF). (“Review of the National Programme on the Development of Family Medicine 2011-2015 in Tajikistan” 2016)	FIN3. Financial protection in PC, FIN4. Comprehensiveness of financial protection for PC services	Surveys were conducted between 2008 and in 2013, in order to explore the impact of the basic benefits package on the financial burden of the population (HPAU, 2013c). Evaluations found a reduction in under-the-table payments and increased formal salaries of physicians. However, overall OOP costs only decreased slightly (Jakab et al. 2008; Bobokhojaeva et al., 2009). There is general agreement that the basic benefit package is not fully financed. One of the challenges is that the introduction of the basic benefit package was not accompanied by changes in budget planning principles and methods. Furthermore, the levels and methods for establishing exemptions to co-payments are inadequate. Specified social groups and patients with certain diseases have been exempted from co-payments (Table 3.3). However, the percentage of the population exempt from co-payments in the rayons where the guaranteed benefit package is being piloted is very small, constituting, for example, only 4% of the population in Spitamen rayon. (Khodjamurodov et al. 2016)
 
The basic benefit package should be further revised and harmonized in relation to current copayment schemes to explore inequities, especially for identified population groups and geographic areas. . (“EVALUATION OF THE STRUCTURE AND PROVISION OF PRIMARY CARE IN TAJIKISTAN” 2014)
 
An internal evaluation of the Ministry of Health on the impact of the BBP conducted in 2008 at the hospital level comparing trends before and after the introduction of BBP in pilot and control districts has shown no reduction in the overall patient financial burden with utilising services, with the exception of deliveries and exempt patients  . (Schwarz et al. 2013)
 
The time series analysis of four surveys conducted between 2005 and 2011 in rural Tajikistan shows that across the time-span the practice of informal payments has not decreased, and out-of-pocket expenditures on medicine has doubled. This observation comes in contradiction with the national efforts to strengthen the current health financing reforms towards equitable and affordable access to primary health care services and international calls for universal coverage. In 2011, there were differences across the geographical regions of Tajikistan in out-of-pocket expenditures, as well as across the relative economic groups. The fact that 'better-off' patients report paying substantially more and in higher proportions hints towards a discrimination from providers along the capacity to pay of Patients.  Thus, it can be concluded that if the main aim of the health reforms is to improve the performance of the health system in offering affordable and equitable health care services, the financial barriers that patients face when utilising PHC must be addressed. In fact, the BBP policy alone proves not to be a sufficient instrument if not accompanied by parallel efforts to improve delivery of PHC services such as incentives for family doctors to deliver quality care, improved prescription rationality, and measures to guarantee an affordable and equitable drug supply system. Such measures require increased public investments in the health sector, as seen in neighbouring countries. . (Schwarz et al. 2013)
 
The BBP policy alone proves not to be a sufficient instrument if not accompanied by parallel efforts to improve delivery of PHC services such as incentives for family doctors to deliver quality care, improved prescription rationality, and measures to guarantee an affordable and equitable drug supply system. Such measures require increased public investments in the health sector, as seen in neighbouring countries. . (Schwarz et al. 2013)	
7	High private, Out of Pocket (OOP) and informal expenditure, as a percent of Total Health Expenditure (THE).	Private out-of-pocket (OOP) payments as a percentage of total health expenditure was one of the highest in the WHO European Region, reaching 60.1% . (Khodjamurodov et al. 2016)	FIN4. Comprehensiveness of financial protection for PC services	A basic benefit package was adopted in principle in 2007, with the aim of defining which services should be provided at no cost (focused on essential primary and emergency care) and formalizing additional payments for others (as opposed to current informal payments). However, it is still in pilot mode and has so far only been extended to 14 of the country's 65 districts. .  (Khodjamurodov et al. 2016)	FIN3. Financial protection in PC, FIN4. Comprehensiveness of financial protection for PC services	Private expenditures continue to represent the highest share of total health expenditure; in 2012, out-of-pocket expenditures accounted for 62.5% of total health expenditures (MOH 2014a) (“Review of the National Programme on the Development of Family Medicine 2011-2015 in Tajikistan” 2016)
 
At present, the state budget does not fully cover costs for the provision of services under the BBP in pilot areas. In the 2012 Demographic and Health Survey, 45% of women aged 15-49 reported getting money for treatment as a barrier to accessing health services. Financial constraints are of particular concern for the lowest wealth quintile where 69.1% of women mentioned the lack of financial resources as a problem compared to 30.8% in the highest wealth quintile. Analysis of utilization rates confirm that lower socioeconomic groups make fewer health visits and a third of households have family members who delay or do not seek help for financial reasons. (“Review of the National Programme on the Development of Family Medicine 2011-2015 in Tajikistan” 2016)
 
The very high share of private OOP payments as a percentage of total health expenditure undermines a range of health system goals. Many patients, particularly among poorer groups of the population, simply cannot afford the care they require. In 2011, 26.7% of households in the lowest consumption quintile faced catastrophic expenditure (defined as OOP spending on health that exceeds 40% of a household's non-subsistence spending).  . (Khodjamurodov et al. 2016)
 
Being governmental employees, family doctors working at PHC level earn low wages. They were estimated to range between US$ 123 and US$ 153 per month in 2013 such that workers often rely on informal payments and in-kind contributions to earn additional Income . (Donadel et al. 2016)
 
Public funding for the purchase of medicines has declined significantly since 1991, but the cost of pharmaceuticals, most of which are imported, has increased substantially. In 2013, only 2.8% of government expenditure on health was spent on pharmaceuticals, in both inpatient and outpatient care. This contrasts with 13% of total (mostly public) health expenditure being spent on pharmaceuticals in 1991. The largest share of household OPE for health is dedicated to pharmaceuticals .  (Donadel et al. 2016)
 
Informal, under-the-table payments are very common in Tajikistan and prevail over formal payments in the private sector, the state-run “self-financing health care centres” and the pilot rayons covered by the basic benefit package. Informal payments are made directly as OOP outlay. In a survey conducted in 2010, 39% of respondents reported to have made informal payments, an increase from 33% in 2006 (Diagne, Ringold & Zaidi, 2012). Household surveys in four rural rayons (Dangara, Varzob, Shahrinav and Tursunzade) in 2005, 2007, 2008 and 2011 found that OOP expenditure for formally free primary health care was common, with the median amount increasing from US$ 5.3 in 2005 to US$ 10.7 in 2011. Expenditure on pharmaceuticals represented the biggest financial burden. . (Khodjamurodov et al. 2016)
 
Copayments present barriers to accessing care and were widely reported by patients mostly related to drugs prescribed by FDs or DPs but also to pay for a visit to a medical specialist (after referral) or even to the FD or DP. Copayments were cited as a reason to abstain from or delay a visit to a doctor by a quarter of patients. . (“EVALUATION OF THE STRUCTURE AND PROVISION OF PRIMARY CARE IN TAJIKISTAN” 2014)
 
Efforts have been made to redistribute funds from the health care budget towards primary care, but goals have not yet fully been achieved, probably as a result of underfunding of the health system and ineffective budget allocation. The largest part of total health expenditure is private: out-of-pocket payments accounted for almost two thirds of total expenditure in 2012, mainly for pharmaceutical drugs. Access to health services continues to be limited. . (“EVALUATION OF THE STRUCTURE AND PROVISION OF PRIMARY CARE IN TAJIKISTAN” 2014)
 
Adjusted for inflation, OPE for primary care have substantially increased in the period 2005 to 2011. While the proportion of patients reporting the payment of informal consultation fees to providers and their amount were constant over time, the proportion of patients reporting expenditures for drugs has increased, and the median amounts have doubled from 5.3 US$ to 10.7 US$. Thus, the expenditures on medicine represent the biggest financial burden for patients accessing a primary care facility. Regression models showed that in 2011 patients from the most remote district with spread-out villages reported significant higher expenditures on medicine. Besides the steady increase in the median amount for OPE, the proportion of patients reporting making an informal payment to their care provider showed great variations across district of residence (between 20% and 73%) and economic status (between 33% among the 'worst-off' group and 68% among the 'better-off' group). OPE – especially in relation to expenditures for drugs – have increased over time, and vary substantially across geographical areas and economic status. The fact that better-off households report disbursing more and in higher proportions hints towards a discrimination along the capacity to pay from providers. Increased public investments in the health sector, incentives for family doctors to provide PHC services free of charge and a strengthened drug control and supply system are necessary strategies to improve access of patients to services.  .  (Schwarz et al. 2013)
 
Informal payments are not apprehended in this paper as a problem of corrupt practices, but as a symptom of the general condition of the Tajik health system that is under-financed and that lacks efficient and strong policy Instruments. The findings of this analysis show that the overall expenditures of primary health care patients in four study districts in Tajikistan, adjusted for inflation, have doubled between 2005 and 2011. This observation is mainly explained by an increase in the expenditures on medicine. In fact, not only do patients spend more money on prescribed drugs, but they are being prescribed and they obtain their medicine in larger proportions. .  (Schwarz et al. 2013)
 
Four surveys conducted between 2005 and 2011 in rural Tajikistan shows that across the time-span the practice of informal payments has not decreased, and out-of-pocket expenditures on medicine has doubled. This observation comes in contradiction with the national efforts to strengthen the current health financing reforms towards equitable and affordable access to primary health care services and international calls for universal coverage. In 2011, there were differences across the geographical regions of Tajikistan in out-of-pocket expenditures, as well as across the relative economic groups. The fact that 'better-off' patients report paying substantially more and in higher proportions hints towards a discrimination from providers along the capacity to pay of patients. . (Schwarz et al. 2013)
 
In a context where OPE are important, drugs represent an important income source for health service providers. Such a situation does not favour rational prescribing nor efficient service delivery, and is potentially harmful for patients. In particular, the economic ramifications cause high levels of expenditure for patients and households with detrimental, knock-on effects in the more vulnerable segments of the population. Analysis of the survey we conducted in 2014 in rural and semi-urban Tajikistan showed a high rate of drug prescription, an irrational use of antibiotics and vitamins and the common use of injections to administer medicines at the PHC level. Expenditures for drugs represented more than three-quarters of the total amount paid in the course of a visit to a PHC provider. It resulted that a third of the interviewees who did not obtain the drugs prescribed explained it to their inability to pay .  (Donadel et al. 2016)	
8	Poor health system allocative efficiency.	Apart from its quality of universal access, the organisation of the Soviet (Tajik) health system ran counter to the principles of Primary Health Care. The system was hierarchical and centralised, resources were concentrated on treatment at hospitals rather than prevention or outpatient treatment, and the workforce was highly specialised, rather than making effective use of generalist physicians and nurses . (Parfitt and Cornish 2007)	GOV2. Accountability arrangements	The Strategic Plan for the Rationalization of the Health Care Facilities Network of the Republic of Tajikistan for 2011–2020 envisages that the number of oblast, rayon, city and rural hospitals will be reduced by 30% by 2020, whereas the number of primary health care facilities is envisaged to increase (HPAU, 2013b). . (Khodjamurodov et al. 2016)
 
The Joint Decree of the Ministry of Health and Social Protection and the Ministry of Finance “on the management and financing structure of primary care facilities in the Ministry of Health System of the Republic of Tajikistan” (adopted in June 2008) envisaged that at least 40% of city or rayon budgets should be allocated to primary health care (Egamov, Bogadyrova & Akkazieva, 2014c).  . (Khodjamurodov et al. 2016)	GOV2. Accountability arrangements	As the allocation structure of overall public funds indicates, this goal has not yet been reached, probably because of the overall underfinancing of the health system and the ineffective mechanisms of budget allocation and provider payment (HPAU, 2013b). . (Khodjamurodov et al. 2016)
 
While it remains low in absolute and relative terms, the structure shows a favourable trend towards increased spending in PHC. For example, hospital expenditure has decreased to 53% and PHC expenditure has increased to 37% in 2014 (versus 65% and 23% in 2007, respectively). This change can be attributed to the adoption of the joint decree by the MOH and MOF in June 2008 on the management and financing structure of primary care facilities. According to this decree, at least 40% of the rayon/ city health budget is to be allocated to PHC.  . (“Review of the National Programme on the Development of Family Medicine 2011-2015 in Tajikistan” 2016)
 
Efforts have been made to redistribute funds from the health care budget towards primary care, but goals have not yet fully been achieved, probably as a result of underfunding of the health system and ineffective budget allocation. The largest part of total health expenditure is private: out-of-pocket payments accounted for almost two thirds of total expenditure in 2012, mainly for pharmaceutical drugs. Access to health services continues to be limited. . (“EVALUATION OF THE STRUCTURE AND PROVISION OF PRIMARY CARE IN TAJIKISTAN” 2014)
 
The existing management structure for the majority of public providers is characterized by a vertical hierarchy and inflexible financing mechanisms that favour hospital over primary health care and result in an inefficient use of scarce resources. . (Khodjamurodov et al. 2016)
 
Most public health expenditure (54% in 2013) was spent on inpatient care, with only 34.8% in 2013 being allocated to primary health care. . (Khodjamurodov et al. 2016)
 
The process of budget formation in Tajikistan continues to be based on inputs (in particular the number of beds and health workers) rather than outputs (per capita financing for primary health care or case-based payments for inpatient or specialized health services). This perpetuates the incentives for overcapacity and emphasizes structure over content and quality of care. (Khodjamurodov et al. 2016)	
9	Weak funding mechanisms for primary care facilities.	Budgets of health facilities are generally determined on the basis of past expenditures and inputs . (Khodjamurodov et al. 2016)
 
For both primary and inpatient care, planning continues to follow mechanisms inherited from the Soviet period, with an emphasis on inputs and staffing rather than on quality and outputs (although there are pilots of per capita financing for primary health care, see section 3.7). While health reforms were introduced in Tajikistan in 2002 with the aim of moving towards a financing system based on activities or the size of the population covered, until recently the formation of health budgets was still highly centralized and based on inputs. The Ministry of Health and Social Protection has now recognized that the standardized budget lines for inpatient care provide incentives for overcapacity and a too extensive structure of health facilities, while ignoring the content and quality of the care provided (Ministry of Health and Social Protection, 2014). . (Khodjamurodov et al. 2016)	FIN1. Primary care expenditure 	A new population-based budget formation has been piloted in primary health care since 2013 in an effort to move away from the normative-based budget formation characteristic of the Soviet period and to improve the equity and efficiency of public expenditure on health. The Health Services Improvement Project (expected closing date in 2019 ) piloting the use of capitation payment and performance-based incentives in primary health care facilities. . (Khodjamurodov et al. 2016)
 
The allocation of funds to PHC providers based on full capitation (applies to the whole health facility budget) has been piloted since 2013 with the support of Development Partners. This was first piloted in Sughd oblast and is planned for rollout across the country in coming years. Furthermore, between 2014 and 2015, the budget formation for PHC centres was revised by applying a capitation formula. It is planned that, in 2017, the budget for PHC will be formed based on capitation and should be increased to allow improved access and quality in PHC. In parallel, a new incentive mechanism of results-based financing for PHC has been piloted since 2013. This mechanism should strengthen human resource capacity, infrastructure and technical resources in facilities, contributing to the quality of health services. . (“Review of the National Programme on the Development of Family Medicine 2011-2015 in Tajikistan” 2016)	FIN1. Primary care expenditure , FIN2. Payment methods in PC , MAN2. PC facility budgets	Currently, per-capita financing for PHC is applied only to the non-salary proportion of the overall budget, which represents only about 10% of total PHC spending. Expansion to full per-capita financing for PHC services in Tajikistan is currently being supported by Development Partners and piloted in one rayon. The large majority of public funds are allocated to PHC facilities via line-item budgets that do not provide incentives for quality. . (“Review of the National Programme on the Development of Family Medicine 2011-2015 in Tajikistan” 2016)	
11	Weak infrastructure and equipment.	Most health facilities in Tajikistan were constructed in the period 1938–1980, and their condition has deteriorated sharply since the country's independence, mainly through a lack of investment in reconstruction or the purchase of new equipment. Since 1990, there has been little investment in modern medical equipment for rayon and oblast  hospitals, while the remaining equipment fell into a state of disrepair. Where investments took place, they were mostly directed at large national-level health facilities in Dushanbe, as well as areas of health care that benefited from donor assistance, such as tuberculosis (supported by KfW and the Global Fund) and mother and child health (supported by KfW). The poor material conditions of many health facilities undermine access to health services, quality of care, and staff and patient satisfaction. . (Khodjamurodov et al. 2016)
 
 
Tajikistan's health infrastructure has suffered from the effects of the civil war and decades of underinvestment. External donors have provided some assistance to remedy this, but basic necessities (such as heating, water, sanitation and electricity) are still lacking in many health facilities. Medical equipment is often outdated or lacking altogether. . (Khodjamurodov et al. 2016)	DGN1. Laboratory , DGN2. Imaging, STR 1. Basic amenities , TCH1. Basic technology	An effort to improve the accessibility and full use of equipment, capacity, diagnostics and treatment practices for family medicine specialists in PHC has been made during implementation of the Family Medicine Programme. . (“Review of the National Programme on the Development of Family Medicine 2011-2015 in Tajikistan” 2016)
 
The MOH has also put an emphasis on the standardization of infrastructure in PHC facilities, including equipment and regular maintenance. In recent years, renovations of PHC facilities (rayon health centres, rural health centres and health houses) were undertaken. Furthermore, in 2013, the MOH established a Centre on Medical Device Maintenance in order to repair and maintain equipment in health facilities procured or received from Development Partners. This Centre, however, is not yet fully operational as the legislative base has not been fully developed. . (“Review of the National Programme on the Development of Family Medicine 2011-2015 in Tajikistan” 2016)
 
The National Health Strategy for the period 2010-2020 emphasizes the importance of PHC by allocating additional funds for PHC; at least 40% of the rayon/city budgets have to be allocated to PHC (see Objective 4.3). Additionally, the MOH ensures access to PHC services via the basic benefits programme (BBP) or Decree No. 600. PHC facilities may purchase needed drugs and equipment or refurbish their facilities using funds collected from these two interventions. . (“Review of the National Programme on the Development of Family Medicine 2011-2015 in Tajikistan” 2016)
 
In 2005, with the support of Development Partners, a reproductive health logistics management and information system (LMIS) was introduced . (“Review of the National Programme on the Development of Family Medicine 2011-2015 in Tajikistan” 2016)	DGN1. Laboratory , DGN2. Imaging, FIN1. Primary care expenditure , GOV2. Accountability arrangements, GOV4. Quality assurance mechanisms, STR 1. Basic amenities , TCH1. Basic technology	Tajikistan's health infrastructure has suffered from the effects of the civil war and decades of underinvestment. External donors have provided some assistance to remedy this, but basic necessities (such as heating, water, sanitation and electricity) are still lacking in many health facilities. Medical equipment is often outdated or lacking altogether. (Khodjamurodov et al. 2016)
 
Lack of equipment, devices and aids has also been reported at the level of primary health care, where family doctors and rayon physicians consistently report being insufficiently equipped or lacking altogether the necessary laboratory and radiography facilities (WHO Regional Office for Europe, 2014a). . (Khodjamurodov et al. 2016)
 
Quality of care is a major concern in Tajikistan for a number of reasons. The material conditions in many health facilities have suffered from years of underinvestment, and available technology and equipment are often outdated and obsolete. . (Khodjamurodov et al. 2016)
 
Across the country, warehouses, logistic chains, computer-based systems and trained personnel are now in place and ensure the effective management of logistics and information for reproductive health services in primary care. . (“Review of the National Programme on the Development of Family Medicine 2011-2015 in Tajikistan” 2016)
 
Availability of laboratory services and X-ray facilities is poor: one third of FDs and DPs have no or insufficient access to microbiological laboratory services and one quarter no or insufficient access to X-ray. Observed interregional differences are wide. . (“EVALUATION OF THE STRUCTURE AND PROVISION OF PRIMARY CARE IN TAJIKISTAN” 2014)
 
Capital investment in the health system has been negligible since Tajikistan's independence. Funds for rehabilitation of existing buildings or construction of new ones have been lacking, and modern equipment tends to be obsolete  and dysfunctional. In most health facilities, heating, water supply, sewage systems, sanitation, electricity and communication systems are unsatisfactory. Lack of equipment, devices and aids has also been reported at the level of primary health care, where family doctors and rayon physicians consistently report being insufficiently equipped or lacking altogether the necessary laboratory and radiography facilities (WHO Regional Office for Europe, 2014a). (HiT Report) . (Khodjamurodov et al. 2016)	
12	Weak regulation and governance of pharmaceuticals.	A list of essential drugs was introduced in 1994 and is revised regularly. However, most pharmacists and physicians are unaware of the essential drug list and do not use it in their practice. Even state entities such as Pharmacon, Sogd Pharmacy and Khatlon Pharmacy cannot secure the supply of the drugs on the essential drug list, do not follow the drug selection principles and import a large range of other drugs. . (Khodjamurodov et al. 2016)	MED1. Essential medicine list , MED2. Generic prescribing	Almost all state pharmacies have been privatized. . (Khodjamurodov et al. 2016)
 
An Essential Drugs List has been adopted and is regularly updated. Procurement of drugs and medical supplies is carried out in conformity with the law “on state procurement of goods, works and services”. To establish mechanisms for the procurement of medicines and medical supplies that meet the needs of the population, the Republican Centre for Pharmaceutical and Medical Equipment Services was established (Ministry of Health and Social Protection, 2014). Among other things, the Centre carries out the importation of drugs on the basis of the Essential Drug List, which was developed in conformity with international standards, with costs that are 15–20% less than alternative pharmaceuticals (Ministry of Health and Social Protection, 2014). . (Khodjamurodov et al. 2016)
 
In addition, the Unit on State Procurement of Goods, Works and Services was established in the Department of Pharmaceuticals and Medical Goods under the Ministry of  health and Social Protection. The Unit is responsible for the procurement of pharmaceuticals from the state budget through a national bidding process. Health facilities at  oblast and municipal levels procure drugs and medical goods through the Government Agency for State Procurement of Goods, Works and Services (Ministry of Health and  Social Protection, 2014). Pharmaceuticals for the treatment of tuberculosis, HIV/AIDS, malaria and diabetes, which the country has received free of charge until 2015 under agreements with international development partners (Ministry of Health and Social Protection, 2012). . (Khodjamurodov et al. 2016)	EFC1. Unnecessary procedures, EFC2. Prescription , MED1. Essential medicine list , MED2. Generic prescribing	Quality of medicines. Reporting acknowledges impressive progress in recent years to improve the regulation of medicines, including the introduction of an agency for surveillance of the pharmaceutical market. There is, however, a need to increase capacity in laboratories and inspection functions in services delivery, as well as to develop a comprehensive registration system for medicines. There is also no functioning system of pharmacovigilance in place, compromising the extent to which side-effects, low effectiveness and quality of medicines are reported. . (Khodjamurodov et al. 2016)
 
While a national essential drug list is in place, the absence of price regulation and pricing policies poses challenges for procurement . (“Review of the National Programme on the Development of Family Medicine 2011-2015 in Tajikistan” 2016)
 
There has also been an increase in the overall number of pharmacies, as well as an increase in the numbers of low-quality medicines and new drugs unknown to the majority of health professionals of the country. The widespread trafficking and availability of counterfeit pharmaceuticals is a major area of concern. . (Khodjamurodov et al. 2016)
 
Public funding for the purchase of medicines has declined significantly since 1991, but the cost of pharmaceuticals, most of which are imported, has increased substantially. In 2013, only 2.8% of government expenditure on health was spent on pharmaceuticals, in both inpatient and outpatient care. This contrasts with 13% of total (mostly public) health expenditure being spent on pharmaceuticals in 1991. The largest share of household OPE for health is dedicated to pharmaceuticals .  (Donadel et al. 2016)
 
As Khodjamurodov and Rechel have reported, “although much of the discussion on out-of-pocket payments in Central Asia has focused on informal payments, expenditures on outpatient drugs, which always have been legal and required, might be more important in total magnitude and frequency than informal payments”. In fact, expenditures on medicine have shown to be a large and increasing share of the total health expenditure of households  .  (Schwarz et al. 2013)
 
In Tajikistan, doctors at PHC level seem to overprescribe drugs, in view of the number of drugs prescribed concomitantly and the high level of polypharmacy. It can be explained by the low official salary FD earn from the government, estimated to range between US$ 123 and US$ 153 per month in 2013 which is not enough to cover essential needs. As a consequence, doctors often rely on informal payments and in-kind contributions from patients. Expenditures for drugs represented 2.8 % of governmental health budget in 2013 so that pharmaceuticals are mainly financed by patients through informal OPE (both at hospital and primary care level). Such conditions do not favour rational prescribing as the prescription of a high number of drugs can represent a complementary income source to doctors and pharmacists. .  (Donadel et al. 2016)
 
However, the predominant role of the private sector and the underdeveloped regulatory environment have led to an underuse of generic drugs over the delivery of brand names. Already in 2005, the inclusion of 139 brand names in the Tajikistan Essential Drug List was a source of concern for the WHO, suggesting that the Tajik government had not embraced the promotion of generic drugs, and instead allowed the entry of expensive drugs into the market which the country could not afford. Although the situation has improved since then, the lack of evidence-based guidelines, the insufficient education of doctors and pharmacists, and the pressure from the pharmaceutical industry all result in irrational prescribing practices. The prescription of brand names instead of International Nonproprietary Names (INN) for pharmaceutical substances means that patients purchase expensive drugs instead of generics, ultimately impairing access of poor households to health care.  . (Donadel et al. 2016)
 
Analysis of the survey we conducted in 2014 in rural and semi-urban Tajikistan showed a high rate of drug prescription, an irrational use of antibiotics and vitamins and the common use of injections to administer medicines at the PHC level. Expenditures for drugs represented more than three-quarters of the total amount paid in the course of a visit to a PHC provider. It resulted that a third of the interviewees who did not obtain the drugs prescribed explained it to their inability to pay. .  (Donadel et al. 2016)
 
In the Tajik context where OPE are important, drugs represent an important income source for various health system players, principally pharmacists incentivized to deliver drugs without prescription to increase their salary. Such situations do not favour rational prescribing nor efficient service delivery, and are potentially inappropriate or even harmful for patients. Patients' and households' interests are put at risk, especially the more vulnerable segments of the population. . (Donadel et al. 2016)
 
Informal payments are not apprehended in this paper as a problem of corrupt practices, but as a symptom of the general condition of the Tajik health system that is under-financed and that lacks efficient and strong policy Instruments. The findings of this analysis show that the overall expenditures of primary health care patients in four study districts in Tajikistan, adjusted for inflation, have doubled between 2005 and 2011. This observation is mainly explained by an increase in the expenditures on medicine. In fact, not only do patients spend more money on prescribed drugs, but they are being prescribed and they obtain their medicine in larger proportions. .  (Schwarz et al. 2013)	
13	Weak health information management systems.	Documentation_required	INF1. Data capture, INF2. Aggregation of data	Since 2014, a number of computers were distributed among health care facilities to improve data collection and introduce electronic submission of statistical reports, using the unified District Health Information Software (DHIS-2). (Khodjamurodov et al. 2016)
 
A strategic development plan for the health information system has been drawn up, envisaging a unified health information system. As part of the implementation of this plan, reporting forms have been updated and improved. In 2012, all health facilities were ordered by the Ministry of Health and Social Protection to introduce the reporting programme “Medstat” (Ministry of Health and Social Protection, 2013a). The project “Technical assistance to support the strengthening of the health information system in Tajikistan” (2012–2016), funded by the European Union (EU), aims to strengthen the health management information system for the Ministry of Health and Social Protection, the Ministry of Justice, the Agency for Statistics and the Civil Registration Office. It prepares for the countrywide introduction of DHIS-2 for data entry, analysis and reporting. . (Khodjamurodov et al. 2016)	INF1. Data capture, INF2. Aggregation of data	Poor training of staff and the absence of modern information technologies are obstacles to reliable data collection. Forms continue to be completed manually, making the processing and analysis of data cumbersome. A survey of 255 family doctors and 225 rayon physicians in 2012 found that 90% of participating family doctors and 82% of rayon physicians did not use a computer in their practice; only 2–4% of those who did reported using a computer used it for keeping patient records (WHO Regional Office for Europe, 2014a). In 2013, only 30.7% of health facilities in 20 rayons reported using DHIS-2 (Ministry of Health and Social Protection, 2014). . (Khodjamurodov et al. 2016)
 
Within the framework of the project, equipment has been distributed in all rayons and cities and around 800 specialists from the Ministry of Health and Social Protection and the Civil Registration Office had been trained by October 2014. Utilization of the DHIS-2 software has started on a pilot basis and was anticipated to be rolled out countrywide in 2015. . (Khodjamurodov et al. 2016)
 
Availability of timely and accurate services delivery data. Despite monitoring and evaluation processes in place for PHC facilities, strengthening recording, reporting and actions for quality improvement are of critical importance. Developing indicators in line with the process of approving new CGPs (recall Priority 1) has real potential to streamline the process of identifying measures and ensuring these are in accordance with evidence-based practices. The paper-based process in place has previously been described as time consuming and a possible source of duplication (Jean-Richard 2010; Akkazieva et al. 2015). . (“Review of the National Programme on the Development of Family Medicine 2011-2015 in Tajikistan” 2016)
 
Patients' clinical records are well kept by doctors and nurses in primary care, but are paper-based as a rule, as computers are not available. . (“EVALUATION OF THE STRUCTURE AND PROVISION OF PRIMARY CARE IN TAJIKISTAN” 2014)	
15	Outdated education and training standards for the specialty of family medicine and general practice.	Documentation_required	WRK4. Academic status of PC	Reforming medical education to bring it in line with international standards and structures has been one of the key directions of health reform. General practice (family medicine) has been established as a medical specialty and professional and training or retraining courses have been implemented for both physicians and nurses. . (Khodjamurodov et al. 2016)
 
Tajikistan has started to bring its university education in line with the Bologna process. . (Khodjamurodov et al. 2016)
 
A post-university specialty-training program (PUST) in family medicine was developed and is being piloted.  . (“Review of the National Programme on the Development of Family Medicine 2011-2015 in Tajikistan” 2016)
 
Development of a family medicine workforce was initiated with the introduction of a six month retraining programme for family doctors since 2001. In 2015, the Family Medicine Centre, regional centres and inter-rayon departments retrained 580 health workers (171 doctors, 409 nursing staff) in family medicine, with a further 274 specialists (23 doctors and 251 nurses) currently undergoing trainings. In 2014, TSMU had 90 graduates specialized in family medicine. In 2015, medical colleges across the country had 575 graduates in general medicine with a specialization in family medicine. . (“Review of the National Programme on the Development of Family Medicine 2011-2015 in Tajikistan” 2016)
 
The Ministry of Health and Social Protection has limited the number of new students per year in order to improve the quality of the training provided to students and to avoid a surplus of staff. . (Khodjamurodov et al. 2016)
 
Undergraduate medical education at Tajik State Medical University (TSMU) has undergone reforms working to increase hours of clinical practice. The six-year undergraduate education programme provided by TSMU has been revised, incorporating new learning objectives and teaching models into the curriculum. To date, the first five years of the curriculum have been adapted, with a significant reduction in taught hours and increased focus on developing competencies through practice-based learning. . (“Review of the National Programme on the Development of Family Medicine 2011-2015 in Tajikistan” 2016)
 
General practice (family medicine) and general practitioners were included in the list of medical professions in 1998. Departments of family medicine have been established at the Tajik State Medical University and at eight medical education centres throughout the country. Family doctors are trained at the graduate and postgraduate level. Graduate training is provided by the Tajik State Medical University named after Abuali Ibn Sina (TSMU) within a six-year curriculum. For specialists in family medicine, clinical internship is one year, and clinical residency is two years. Postgraduate training is provided by the Tajik Institute of Postgraduate Medical Training (HPAU, 2013e). . (Khodjamurodov et al. 2016)
 
Training programmes for family medicine doctors and nursing staff are developed and approved on priority health issues (for example, respiratory diseases, ischemic heart diseases, emergency health care and organization of palliative care by family nurses). The Postgraduate Medical Institute (PGMI) supports the development of curricula, trainings and TOT for nursing practice. . (“Review of the National Programme on the Development of Family Medicine 2011-2015 in Tajikistan” 2016)
 
Repair and equipping of training facilities. Advancements in the resourcing of training facilities, including an established clinical skills centre, have been achieved. . (“Review of the National Programme on the Development of Family Medicine 2011-2015 in Tajikistan” 2016)	WRK4. Academic status of PC	Family medicine continues to suffer from low prestige, working conditions tend to be poor and most medical graduates choose other specialties. . (Khodjamurodov et al. 2016)
 
Institutions for the training of health workers generally lack appropriate training materials, equipment and infrastructure, as well as sites for practical experience and qualified teachers. . (Khodjamurodov et al. 2016)
 
Developing a registration system in Tajikistan has great importance with respect to monitoring, planning and generating opportunities for continuous performance improvement. . (“Review of the National Programme on the Development of Family Medicine 2011-2015 in Tajikistan” 2016)
 
The current method of licensing for family doctors in Tajikistan has remained the same as that used during the Soviet period. Updating and systematizing licensing to practice is critical as a guarantor of competencies  following initial education (Langins and Borgermans 2015). Improving this process is also of particular importance for continuous  medical education, regulation of professional regulatory bodies and associations, and the overall management and control of entry, exit and recertification of health professionals. . (“Review of the National Programme on the Development of Family Medicine 2011-2015 in Tajikistan” 2016)
 
The continuous updating and modernization of the family medicine curriculum is necessary for the uptake of best available evidence into formal education. Bringing further alignment between training and clinical practice is also of critical importance for strengthening and sustaining a competent health workforce. The possibility to incorporate a year of full immersion in clinical practice into the current undergraduate curriculum for the final (sixth) year of study is reportedly being explored by TSMU. . (“Review of the National Programme on the Development of Family Medicine 2011-2015 in Tajikistan” 2016)
 
For interns and residents, developing paid postgraduate training positions and giving interns and residents supervised access to patients have been noted as relevant measures to improve perception of family medicine practice. . (“Review of the National Programme on the Development of Family Medicine 2011-2015 in Tajikistan” 2016)
 
Promoting family medicine specialty at the pre-graduate level. Students should be regularly exposed to family medicine practice during their entire six years of undergraduate medical education to become familiar with this area of practice. However, without a clear policy to give priority to family medicine, the promotion of family medicine practice will remain very difficult. Known mechanisms to incentivize a positive and rewarding working environment in family medicine should be systematically implemented. . (“Review of the National Programme on the Development of Family Medicine 2011-2015 in Tajikistan” 2016)
 
Lack of competencies and skills among physicians and nurses are an obstacle to further expanding the scope of services at PHC level and consequently to the realization of reform aims. New approaches in education and teaching should be explored, including distance learning. . (“EVALUATION OF THE STRUCTURE AND PROVISION OF PRIMARY CARE IN TAJIKISTAN” 2014)
 
By April 2013, a total of 8720 health workers had been trained or retrained in family medicine, including 3700 at university level and 5020 at nursing schools (HPAU, 2013e). However, by November 2013, only about 55% of the projected need in doctors trained in family medicine and 44% of the projected need in mid-level health workers trained in family medicine had been met (Ministry of Health and Social Protection, 2013a) . (Khodjamurodov et al. 2016)
 
Registration of health professionals with regulatory bodies following certification or licensing is an important process for effectively managing and maintaining a roster of health professionals. A reliable registration system is also important for ensuring that medical licenses are up-to-date and that requirements for continuous professional development are adhered to. With the continued advancement of undergraduate and postgraduate training in family medicine practice, developing a registration system in Tajikistan has great importance with respect to monitoring, planning and generating opportunities for continuous performance improvement. Moreover, improving a registration system across the health workforce has important potential in ensuring resources are allocated to train health workers in line with population health needs and migration of the health workforce . (“Review of the National Programme on the Development of Family Medicine 2011-2015 in Tajikistan” 2016)
 
One of the challenges is that the prestige of family medicine continues to be low. A survey among medical graduates in 2012 found that only 0.8% had chosen the specialty of family medicine, while 52.2% chose narrow specialties such as obstetrics/gynaecology or surgery and most preferred to work in hospitals in urban areas (HPAU, 2013e). Salary levels are one reason. While the salaries of health workers were increased by 40% in September 2012, the monthly salary of family doctors still only amounted to 513 somoni, which was slightly below the subsistence level of 536 somoni (approximately US$ 112) (HPAU, 2013e). Other challenges include poor working conditions and a lack of medical equipment (WHO Regional Office for Europe, 2014b). Those health workers who do work in family medicine might also face problems in using their working time productively. A qualitative study of 52 randomly selected health workers in family medicine (24 family doctors, 24 family nurses and 4 narrow specialists) from rayon and rural health centres in four rayons, conducted in July–August 2014, found that health workers spent a considerable time (41.1% of their working time over five consecutive days) on administrative tasks (in particular those related to the health information system), to the detriment of patient care (Bratschi et al., 2015). . (Khodjamurodov et al. 2016)
 
The qualifications of health care workers are another constraint and most have no access to modern periodicals and medical literature. In a small-scale study of selected health care providers in rural areas, physicians and midwives scored on average less than 50% of correct answers on 52 knowledge questions about maternity care (Wiegers, Boerma & de Haan, 2011). . (Khodjamurodov et al. 2016)	
16	Weak and outdated standards for education, training and licensing of nurses in primary care.	Documentation_required	WRK4. Academic status of PC	A nursing faculty has also been established at PGMI and basic nursing training can be completed in three years. This can be followed by a one-year specialty training in family medicine nursing or other specialty . (“Review of the National Programme on the Development of Family Medicine 2011-2015 in Tajikistan” 2016)
 
At a policy level, Family Health Nursing is a key plank of Tajikistan's health reforms (MOH, Tajikistan, 2006), but there is no evidence to date on its Implementation. . (Parfitt and Cornish 2007)
 
In Tajikistan, Family Health Nursing has a core role in the government's nursing development strategy. A target of 8600 postgraduate Family Health Nurses have been identified as necessary to satisfy the requirements of the Primary Health Care sector (MOH, Tajikistan, 2006). This target is to be met through two routes. . (Parfitt and Cornish 2007)
 
In 2000, a re-training programme to equip already-qualified nurses to become Family Health Nurses was initiated, which has produced 500 graduates. More significantly, four medical colleges have implemented a new 4-year curriculum for Family Health Nurses, with 700–900 places in total available each year (MOH, Tajikistan, 2006). The first cohort of these students graduated in May 2006. Thus, the initial target is expected to be met within 9–11 years. . (Parfitt and Cornish 2007)	WRK4. Academic status of PC	Nursing is still poorly developed and many nurses are underqualified. Although they constitute the majority of health workers and contribute significantly to the provision of health services, nursing has so far failed to attract sufficient attention. Many nurses carry out a limited number of functions and do not take independent decisions on patient care. There are however some positive developments in the training of nurses. A nursing faculty has been established at the Postgraduate Medical Institute and nurse training has been upgraded to four-year courses. . (Khodjamurodov et al. 2016)
 
Family nurses are being trained in medical colleges and schools in family medicine. . (Khodjamurodov et al. 2016)
 
 The retraining of nurses for practice in family medicine is active in six rayons across the country. Over the past years more than 305 family nurses were retrained, as well as nurse tutors. The retention rate of retrained nurses in these rayons remains high at 96%. . (“Review of the National Programme on the Development of Family Medicine 2011-2015 in Tajikistan” 2016)
 
Nurses in primary care should be more involved in patient care and less in administration. This may alleviate the problem of physician shortages. Nurses can have greater involvement in clinical work than is currently the case in areas such as the management and follow up of patients with non-communicable diseases, providing information and promoting patient empowerment: these are important domains for nurses. Administration can be organized more efficiently and may largely be delegated to other staff. . (“EVALUATION OF THE STRUCTURE AND PROVISION OF PRIMARY CARE IN TAJIKISTAN” 2014)
 
Despite an initially adverse context, in which nurses were considered doctors' assistants, low in status and without a decision-making role, findings suggest that, following their retraining, nurses are working in new ways. They are taking on responsibility for prevention as well as care, taking independent decisions, and working in partnership with physicians. .  (Parfitt and Cornish 2007)
 
Family medicine nurses are not used effectively, with task duplication and varied roles in educating patients. The population's opinion on nurses is regarded as poor and expectations about nurses' ability to treat patients are low (Akkazieva et al. 2015). . (“Review of the National Programme on the Development of Family Medicine 2011-2015 in Tajikistan” 2016)	
17	Weak Continuing Medical Education (CME) standards.	Documentation_required	WRK4. Academic status of PC	Physicians are formally required to undergo continuous professional education, with a one-month refresher course every five years. There are also many continuous professional education courses within the framework of donor-funded projects . (Khodjamurodov et al. 2016)
 
Retraining is provided through a six-month continuing medical education course for physicians and nurses who want to retrain in family medicine. . (Khodjamurodov et al. 2016)
 
Procedures of continuous training of family medicine doctors were approved by decree of the Ministry of Health on 8 November 2012, Order No. 549, setting the precedence for CME. However, this still requires further investment in order to be rolled out and achieved. . (“Review of the National Programme on the Development of Family Medicine 2011-2015 in Tajikistan” 2016)	IMP4. Continuous professional development , WRK4. Academic status of PC	Continuing education opportunities are poor because of a lack of financial resources (Ministry of Health and Social Protection, 2005b) and non-adherence has no consequences for further medical practice . (Khodjamurodov et al. 2016)
 
A survey conducted by the World Bank in Tajikistan in 2011 found that only 38% of hospital physicians and 29% of primary care physicians had received any type of continuous medical training in the previous 12 months (World Bank, 2013). . (Khodjamurodov et al. 2016)	
18	The domain of primary care suffers low prestige and weak professional standards.	Documentation_required	IMP5. Job satisfaction , WRK4. Academic status of PC	The National Health Strategy of Tajikistan 2010-2020 highlighted the development of PHC based on family medicine practice among the main health reform priorities during this period. That same year, the law on family medicine defining regulatory functions, as well as competencies, rights and obligations of family medicine specialists was enacted (Order No. 676, 29 December 2010). The law triggered the development and further approval of the National Programme on Family Medicine development in Tajikistan for 2011-2015. . (“Review of the National Programme on the Development of Family Medicine 2011-2015 in Tajikistan” 2016)
 
General practice (family medicine) and general practitioners were included in the list of medical professions in 1998. Departments of family medicine have been established at the Tajik State Medical University and at eight medical education centres throughout the country. Family doctors are trained at the graduate and postgraduate level. Graduate training is provided by the Tajik State Medical University named after Abuali Ibn Sina (TSMU) within a six-year curriculum. For specialists in family medicine, clinical internship is one year, and clinical residency is two years. Postgraduate training is provided by the Tajik Institute of Postgraduate Medical Training (HPAU, 2013e). . (Khodjamurodov et al. 2016)	FIN1. Primary care expenditure , FIN2. Payment methods in PC , FIN3. Financial protection in PC, GOV1. Primary care priorities , GOV2. Accountability arrangements, GOV3. Stakeholder participation and engagement, WRK1. PC workforce planning, WRK2. Financial status of GPs/FMs, WRK4. Academic status of PC	The existing legislation on the workload of family medicine doctors requires updating. With focus put on PHC-led services across vertical, disease-specific programmes, the role and scope of work for family medicine doctors needs close consideration. Importantly, reviewing the role and scope of practice should look across levels of the health system, anticipating referrals, transitions and care coordination processes. With the rollout of capitation-based financing across the country, clarifying an understanding of provider workload is now relevant more than ever. . (“Review of the National Programme on the Development of Family Medicine 2011-2015 in Tajikistan” 2016)
 
Positioning the family medicine specialty among medical graduates as a preferred area of practice requires targeted efforts to entice and motivate new graduates to work in PHC. Lack of prestige, poor working conditions in rural areas and the broad scope of activities are key factors challenging the recruitment and sustainability of a strong family medicine workforce (Akkazieva et al. 2015). Creating a positive practice environment for family medicine is an important way to ensure a motivated, available and competent health workforce. . (“Review of the National Programme on the Development of Family Medicine 2011-2015 in Tajikistan” 2016)
 
A limited curative service profile among FDs and DPs, limited diagnostic possibilities in PHC and low referral rates to secondary care are indicative of under-demand and unmet needs . (“EVALUATION OF THE STRUCTURE AND PROVISION OF PRIMARY CARE IN TAJIKISTAN” 2014)
 
Development of working standards for specialists of family medicine and professionals delivering specialized services at PHC level . (“Review of the National Programme on the Development of Family Medicine 2011-2015 in Tajikistan” 2016)
 
One of the challenges is that the prestige of family medicine continues to be low. A survey among medical graduates in 2012 found that only 0.8% had chosen the specialty of family medicine, while 52.2% chose narrow specialties such as obstetrics/gynaecology or surgery and most preferred to work in hospitals in urban areas (HPAU, 2013e). Salary levels are one reason. While the salaries of health workers were increased by 40% in September 2012, the monthly salary of family doctors still only amounted to 513 somoni, which was slightly below the subsistence level of 536 somoni (approximately US$ 112) (HPAU, 2013e). Other challenges include poor working conditions and a lack of medical equipment (WHO Regional Office for Europe, 2014b). Those health workers who do work in family medicine might also face problems in using their working time productively. A qualitative study of 52 randomly selected health workers in family medicine (24 family doctors, 24 family nurses and 4 narrow specialists) from rayon and rural health centres in four rayons, conducted in July–August 2014, found that health workers spent a considerable time (41.1% of their working time over five consecutive days) on administrative tasks (in particular those related to the health information system), to the detriment of patient care (Bratschi et al., 2015). . (Khodjamurodov et al. 2016)
 
Nurses in primary care should be more involved in patient care and less in administration. This may alleviate the problem of physician shortages. Nurses can have greater involvement in clinical work than is currently the case in areas such as the management and follow up of patients with non-communicable diseases, providing information and promoting patient empowerment: these are important domains for nurses. Administration can be organized more efficiently and may largely be delegated to other staff. . (“EVALUATION OF THE STRUCTURE AND PROVISION OF PRIMARY CARE IN TAJIKISTAN” 2014)
 
Family medicine nurses are not used effectively, with task duplication and varied roles in educating patients. The population's opinion on nurses is regarded as poor and expectations about nurses' ability to treat patients are low (Akkazieva et al. 2015). . (“Review of the National Programme on the Development of Family Medicine 2011-2015 in Tajikistan” 2016)
 
Despite an initially adverse context, in which nurses were considered doctors' assistants, low in status and without a decision-making role, findings suggest that, following their retraining, nurses are working in new ways. They are taking on responsibility for prevention as well as care, taking independent decisions, and working in partnership with physicians. .  (Parfitt and Cornish 2007)
 
The success of Family Health Nursing has been undermined by a profound lack of resources. Most fundamentally, the inability of the government to pay regular salaries had led to attrition of the workforce. .  (Parfitt and Cornish 2007)
 
The scope of practice and the competencies of family doctors and rayon physicians continue to be restrictive, with limited involvement in treatment and preventive services (WHO Regional Office for Europe, 2014a). A survey of 255 family doctors and 225 rayon physicians in 2012 found that their role as the first contact point for patients was modest, as was their involvement in the treatment of diseases. Their involvement in the provision of medical procedures and preventive services was even lower (WHO Regional Office for Europe, 2014a). Moreover, the role of health professionals such as midwives and nurses is also underutilized and their scope of practice limited (WHO Regional Office for Europe, 2014b). . (Khodjamurodov et al. 2016)	
19	Weak recruitment and retention.	No precise data exist on the numbers of health professionals leaving the country. While medical schools remain able to attract students, retention of graduates at health facilities poses a serious problem, and the health system continues to lose qualified workers (Ministry of Health and Social Protection, 2012). . (Khodjamurodov et al. 2016)
 
The Ministry of Health and Social Protection has also developed a number of strategies to retain health workers in rural and remote areas (see section 4.2.1), using both financial and nonfinancial incentives, but not all local hukumats have taken measures to improve the working and living conditions of health workers, such as through allocating them land or other benefits (Ministry of Health and Social Protection, 2013a) . (Khodjamurodov et al. 2016)	WRK3. PC workforce availability	Documentation_required	WRK3. PC workforce availability	Documentation_required	
20	Weak accreditation standards in primary care settings.	Documentation_required	GOV4. Quality assurance mechanisms	In 2014, the National Centre for Accreditation of Health Care Facilities was established . (Khodjamurodov et al. 2016)
 
In September 2015, the Republican Centre of Health Accreditation was approved as an accreditation authority by the International Society for Quality in Healthcare (ISQua). Staff of the Republican Centre for Health Accreditation have been trained with support of ISQua. In total, 14 specialists participated in practical courses for the accreditation of medical institutions and were trained in the neighbouring country, Kyrgyzstan. . (“Review of the National Programme on the Development of Family Medicine 2011-2015 in Tajikistan” 2016)	GOV4. Quality assurance mechanisms	Tajik State Standard conducts the annual standardization of medical equipment used in large medical facilities. According to current legislation, accreditation is compulsory for all health facilities independent of ownership. In 2014, the National Centre for Accreditation of Health Care Facilities was established at the State Surveillance Service over Medical Activities and Social Protection, as well as a procedure for conducting the accreditation of medical facilities, organizations and enterprises (Government Resolution No. 600 of 9 September 2014). The first accreditation tool was for mother and child health facilities, while the next set of accreditation tools (for multipurpose hospitals) was developed and submitted for approval. The Ministry of Health and Social Protection has approved the Statement of the Steering Council for Accreditation in the Health Sector and established quality improvement committees in large health facilities.(HiT Report) . (Khodjamurodov et al. 2016)
 
To-date, accreditation of five maternity houses has been conducted, certifying the quality of service provision in maternal and child health services, assessed according to the methodology and support of international experts at ISQua. In September 2015, the accreditation standards in maternal and child health services in Tajikistan were certified by ISQua. . (“Review of the National Programme on the Development of Family Medicine 2011-2015 in Tajikistan” 2016)	
21	Weak regulation of quality of care.	Documentation_required	GOV4. Quality assurance mechanisms	In 2008, a State Surveillance Service over Medical Activities was established (now known as State Surveillance Service over Medical Activities and Social Protection). The service is responsible for regulating the quality of medical care in all health facilities irrespective of ownership and including parallel health services, the private sector and providers of alternative medicine. The Ministry of Health and Social Protection has also developed and approved a number of new clinical protocols. In 2013 and 2014, practically all primary health care facilities and large hospitals were  provided with a set of clinical guidelines. . (Khodjamurodov et al. 2016)	GOV4. Quality assurance mechanisms	Implementation [of quality of care standards] remains challenging. . (Khodjamurodov et al. 2016)
 
Quality of care is a major concern in Tajikistan. At all levels of care, there is little emphasis on quality improvement. While the Ministry of Health and Social Protection has recognized the challenge of improving quality of care, embarking on the revision of existing clinical protocols and developing new ones, much more remains to be done. For most conditions or diseases, clinical protocols are still lacking and those that exist are often outdated (HPAU, 2013h). An evaluation of existing guidelines and protocols undertaken by the Evidence-Based Medicine Centre under the Tajik State Medical University found in 2013 that most guidelines and protocols did not consider the best-available evidence, nor did they convey clear clinical pathways (WHO Regional Office for Europe, 2014b). . (Khodjamurodov et al. 2016)
 
The State Surveillance Service for Medical Activities has been responsible for regulating the quality of medical care since 2008, when the Ministry of Health adopted a methodology for developing clinical practice guidelines that includes involvement of key stakeholders. The methodology is based on the internationally accepted Appraisal of Guidelines for Research and Evaluation (AGREE) instrument. The Centre for Evidence-based Medicine has been established at the Tajik State Medical University, but despite this, quality assurance is weakly developed and has not been integrated into the daily practice of health care workers. . (“EVALUATION OF THE STRUCTURE AND PROVISION OF PRIMARY CARE IN TAJIKISTAN” 2014)	
22	 Weak standards for the development of clinical guidelines and protocols for primary care settings.	Documentation_required	GOV4. Quality assurance mechanisms	 The process of developing clinical guidelines and protocols (CGPs), established in 2002, was revised and enhanced in 2013, aiming to reduce fragmentation and duplication in this process and improve the quality of the methodology for developing CGPs.  At the end of 2013, a permanent expert group under the MOH with the role of coordinating the development and approval of CGPs was established (No. 667, 18 November 2013). The group is also responsible for evaluating CGPs against international standards. . (“Review of the National Programme on the Development of Family Medicine 2011-2015 in Tajikistan” 2016)	GOV4. Quality assurance mechanisms	While the Ministry of Health and Social Protection has recognized the challenge of improving quality of care, embarking on the revision of existing clinical protocols and developing new ones, much more remains to be done. For most conditions or diseases, clinical protocols are still lacking and those that exist are often outdated (HPAU, 2013h). An evaluation of existing guidelines and protocols undertaken by the Evidence-Based Medicine Centre under the Tajik State Medical University found in 2013 that most guidelines and protocols did not consider the best-available evidence, nor did they convey clear clinical pathways (WHO Regional Office for Europe, 2014b). . (Khodjamurodov et al. 2016)
 
Despite efforts made to improve the process of developing CGPs, critical gaps compromising quality remain. The current diffusion of responsibility in the process of developing CGPs has posed a considerable degree of variability in the methods and quality of CGPs by topic-specific areas. Further consistency in the drafting and development of CPGs and/or additional technical assistance for actors involved in this process would prove beneficial for enabling a standardized, evidence-based process. Moreover, while the MOH has prioritized the updating and improvement of existing CGPs, many more remain to be done.  While a process of developing and updating the essential medicines list is in place, this is not linked to the development and updating of CGPs. . (“Review of the National Programme on the Development of Family Medicine 2011-2015 in Tajikistan” 2016)	
23	Weak implementation of clinical guidelines and standards in primary care settings.	Documentation_required	GOV4. Quality assurance mechanisms	The Family Medicine Centre is responsible for the implementation of CGPs and the State Supervision Service of Medical Activities (Khadamot) assumes an inspectoral role, overseeing monitoring and evaluation of their use in clinical practice. . (“Review of the National Programme on the Development of Family Medicine 2011-2015 in Tajikistan” 2016)
  
Processes for implementing CGPs in clinical practice and monitoring and evaluating their use in family medicine are in place and over the last five years the MOH has worked to strengthen these. . (“Review of the National Programme on the Development of Family Medicine 2011-2015 in Tajikistan” 2016)
 
Processes nationally for monitoring family medicine practice are in place and coordinated by the Family Medicine Centre. The Family Medicine Centre has developed a form for the monitoring and evaluation of clinical activities in PHC facilities. In 2014, this monitoring was conducted in Muminabad, Khovaling, Vose and Khamadoni rayons within Khatlon Oblast, all rayons within the RRS Oblast, the cities of Khujand, Isfara, Istaravshan, Kanibadam, Isfara and Penjikent within Sughd Oblast and all rayons in GBAO. The aim of this monitoring process is to provide necessary organizational and instructional assistance to facilities as needed. . (“Review of the National Programme on the Development of Family Medicine 2011-2015 in Tajikistan” 2016)
 
Peer review groups (PRGs) have been introduced in family medicine as a practical approach to problem-solving and proactively tackling opportunities for improving clinical processes. With the support of Development Partners, PRGs have been piloted and offer a very valuable experience for quality improvement in family medicine practice. . (“Review of the National Programme on the Development of Family Medicine 2011-2015 in Tajikistan” 2016)
 
Planned quality inspections (audits) in PHC facilities are carried out across the country by external inspectors reporting on adherence to CGPs for family medicine. . (“Review of the National Programme on the Development of Family Medicine 2011-2015 in Tajikistan” 2016)
Processes nationally for monitoring family medicine practice are in place and coordinated by the Family Medicine Centre. The Family Medicine Centre has developed a form for the monitoring and evaluation of clinical activities in PHC facilities. In 2014, this monitoring was conducted in Muminabad, Khovaling, Vose and Khamadoni rayons within Khatlon Oblast, all rayons within the RRS Oblast, the cities of Khujand, Isfara, Istaravshan, Kanibadam, Isfara and Penjikent within Sughd Oblast and all rayons in GBAO. The aim of this monitoring process is to provide necessary organizational and instructional assistance to facilities as needed.  Peer review groups (PRGs) have been introduced in family medicine as a practical approach to problem-solving and proactively tackling opportunities for improving clinical processes. With the support of Development Partners, PRGs have been piloted and offer a very valuable experience for quality improvement in family medicine practice (Khodjamurodov et al. 2016)	GOV4. Quality assurance mechanisms, IMP1. National or regional PC performance assessment , IMP2. Practice level quality improvement mechanisms 	Implementation of guidelines and protocols is hampered by a number of factors, including a lack of local funding for dissemination, limited opportunities for capacity-building among health workers, and lack of structures for monitoring and evaluation (WHO Regional Office for Europe, 2014b). In the survey in the pilot rayons covered by Project Sino, guidelines were often not available in health facilities, with the exception of tuberculosis treatment guidelines (Matthys, 2014). . (Khodjamurodov et al. 2016)
 
By 2014, diagnostic and treatment standards for family medicine were developed for 85 diseases (Box 1), 36 of which were developed prior to 2010. (See full list of CGPs for PHC services in Annex 2). These have been published and printed by the Family Medicine Centre in a book, approved by MOH Order No. 574, 25 June 2014). At the beginning of the following year (January 2015), 12 000 clinical guidelines were distributed to family medicine specialists according to MOH Order No. 574. . (“Review of the National Programme on the Development of Family Medicine 2011-2015 in Tajikistan” 2016)
 
Since 2015, 10 priority diseases have been defined and one indicator for each disease has been developed based on CGPs (see Priority 3, Objective 3.1). In 2015, 87% of PHC facilities at the rayon/city level were reviewed and 78% were found to be in compliance with the CGPs. . (“Review of the National Programme on the Development of Family Medicine 2011-2015 in Tajikistan” 2016)
 
While efforts have been made to inform family medicine doctors on changes to CGPs and disseminate these to PHC facilities, the complete and accurate use of CPGs in practice has yet to be fully achieved. Persisting challenges compromising the success of these efforts include, in particular, a lack of local funding for complete dissemination of new or revised CGPs and an insufficient number of opportunities and variability in approaches taken to inform providers and build capacity. Of particular concern for effective monitoring of CGPs is the absence of a formal process in their development to prepare indicators necessary for assessing effective and accurate use of CPGs in health facilities. The absence of a process to develop indicators poses a significant challenge for auditing and reviewing implementation and, ultimately, a duplication of processes as indicators are prepared post-hoc by actors for implementation and monitoring. . (“Review of the National Programme on the Development of Family Medicine 2011-2015 in Tajikistan” 2016)
 
Many more FDs than DPs have clinical guidelines available and use them frequently: few nurses have guidelines. . (“EVALUATION OF THE STRUCTURE AND PROVISION OF PRIMARY CARE IN TAJIKISTAN” 2014)
 
Tajikistan performed lower on the CPV vignettes than any of the other countries (P<.01). challenged by poor quality clinical care, regardless of practice setting, specialty, or facility type. Like other settings worldwide, we found high quality of care is the exception and not the norm. The lower quality of care scores, as measured by CPV vignettes, observed in primary health care clinics and rural areas compared with hospitals demonstrates a need to target quality improvement efforts geographically, particularly toward primary care diagnosis and treatment. This appears to be especially important for reaching the poor who are likelier to live in rural areas and access care through primary care providers. The notable decay in quality across the care encounter (from history taking to diagnosis) raises important questions about providers' ability to accurately diagnose and treat conditions. Furthermore, the conditions with the lowest scores—multiple NCD risk factors, birth asphyxia, and postpartum hemorrhage—are conditions for which low-cost, widely available treatments can significantly reduce mortality. The low CPV vignette scores identify specific deficiencies that, if remedied, would rapidly improve quality of care and outcomes. . (Peabody et al. 2017)
 
Guidelines and protocols is hampered by a number of factors, including a lack of local funding for dissemination, limited opportunities for capacity-building among health workers, and lack of structures for monitoring and evaluation (WHO Regional Office for Europe, 2014b). In the survey in the pilot rayons covered by Project Sino, guidelines were often not available in health facilities, with the exception of tuberculosis treatment guidelines (Matthys, 2014). . (Khodjamurodov et al. 2016)
 
Overprescribing: There were 1281 (80.1 %) patients who received a drug prescription after visiting a doctor at PHC level. 16.2 % of them had five or more drugs prescribed concomitantly. The number of drugs prescribed to patients ranged from 0 to 8 and was statistically different across regions (RRS region =3.3; Khatlon region = 3.1; p = 0.05), after adjusting for age and sex. In 31.1 % of cases, prescriptions included an intra-venous (IV) injection; in 45.6 % of cases, a non-IV injection; in 52.9 % of cases, an antibiotic; and in 61.0 % of cases, vitamins. Patients suffering from a respiratory disease had higher odds of being prescribed an IV injection and antibiotics. Vitamins were widely prescribed across all diseases. In 94.5 % of cases, the patients interviewed procured at least one of the prescribed drugs. Among those who received a prescription, 2.0 % were not able to procure at least one drug due to a lack of money. In 94.9 % of cases, respondents reported purchasing drugs in private pharmacies. Median expenditures for drugs procured following consultation were 45 TS (US$ 6.9) corresponding to 77.6 % of total expenditures related to the visit (58 TS, US$ 8.8). In a context where OPE are important, drugs represent an important income source for health service providers. Such a situation does not favour rational prescribing nor efficient service delivery, and is potentially harmful for patients. In particular, the economic ramifications cause high levels of expenditure for patients and households with detrimental, knock-on effects in the more vulnerable segments of the population. Analysis of the survey we conducted in 2014 in rural and semi-urban Tajikistan showed a high rate of drug prescription, an irrational use of antibiotics and vitamins and the common use of injections to administer medicines at the PHC level. Expenditures for drugs represented more than three-quarters of the total amount paid in the course of a visit to a PHC provider. It resulted that a third of the interviewees who did not obtain the drugs prescribed explained it to their inability to pay . (Donadel et al. 2016)	
24	Weak performance on the attribute of first contact care.	Documentation_required	ACS1. Accessibility, ACS2. Financial affordability, ACS3. Acceptability	The MOH has defined family medicine doctors as the gatekeeper to health services in Tajikistan, according to the National Health Strategy 2010-2020. To realize this policy, under the BBP a new conditionality was placed on hospitalization: if a patient is referred for hospitalization by a family medicine doctor, then the patient will pay only a co-payment; whereas visiting hospitals directly means services are to be paid in full by the patient. This policy acts as an incentive for people to go to PHC and utilize services there rather than going directly to inpatient care. . (“Review of the National Programme on the Development of Family Medicine 2011-2015 in Tajikistan” 2016)
 
As part of the basic benefit package introduced in 2007, eight co-payment categories were created in the pilot rayons covered by the programme. For each category, the average amount a patient was supposed to contribute was set significantly lower than that reported for under-the-table payments for the same health care intervention. In addition, a 30% (for patients referred from the primary health care level) and 70% (for self-admission without any referral) co-payment differential was introduced. This differential co-payment was intended to strengthen the role of primary health care and to direct the flow of patients to primary health care units rather than hospitals. In 2009, co-payment levels increased to 50% and 80%, respectively. . (Khodjamurodov et al. 2016)	DES1. Referral system , FIN3. Financial protection in PC, FIN4. Comprehensiveness of financial protection for PC services	In 2014, the Health Policy and Analysis Unit (HPAU) carried out a study on access to PHC as part of an analysis of bottlenecks to health system strengthening (Akkazieva et al. 2015). With the introduction of family medicine as a gatekeeper to health care services, the referral system has been improved slightly and reinforced further by the introduction of the BBP. . (“Review of the National Programme on the Development of Family Medicine 2011-2015 in Tajikistan” 2016)
 
Copayments present barriers to accessing care and were widely reported by patients,  mostly related to drugs prescribed by FDs or DPs but also to pay for a visit to a medical specialist (after referral) or even to the FD or DP. Copayments were cited as a reason to abstain from or delay a visit to a doctor by a quarter of patients.  . (“EVALUATION OF THE STRUCTURE AND PROVISION OF PRIMARY CARE IN TAJIKISTAN” 2014)
 
Most patients live within 20 minutes' travel of their FD or DP; more than 40 minutes is exceptional. Most FDs, DPs and nurses work within 5 kilometres of a district hospital.  Patients can generally see their FD, DP or nurse the same day. Only a minority of FDs, DPs and nurses offer weekly opening hours in the evening, but patients are generally satisfied with opening hours. . (“EVALUATION OF THE STRUCTURE AND PROVISION OF PRIMARY CARE IN TAJIKISTAN” 2014)
 
Access by wheelchair is not easily achievable in most practices visited by patients. . (“EVALUATION OF THE STRUCTURE AND PROVISION OF PRIMARY CARE IN TAJIKISTAN” 2014)
 
Although FDs have fewer patients than DPs (1853 versus 1966), the number is still well above the national standard of 1200 in rural areas and 1500 in urban. Staff shortages were reported by almost half of FDs and DPs. Patients have many contacts per year with PHC workers, visiting FDs/DPs nine times and being seen at home 10 times; the respective figures for seeing nurses are 11 and 16. Most patients believe they have no freedom of choice of doctor: they are assigned to their FD or DP. . (“EVALUATION OF THE STRUCTURE AND PROVISION OF PRIMARY CARE IN TAJIKISTAN” 2014)
 
Gatekeeping by family medicine specialists at the primary level continues to be weak. Many patients access higher levels of care directly without referral from primary care and there is a need to strengthen systems of referral and coordination. There is also poor integration of primary and secondary care with regards to the continuity of care. . (“Review of the National Programme on the Development of Family Medicine 2011-2015 in Tajikistan” 2016)
 
FDs and DPs have a limited scope of practice, with a narrow range of curative services offered. Their role in first contact with patients' health problems is modest, as is their involvement in the treatment of diseases, although FDs are involved to a greater extent than DPs. Provision of medical procedures and preventive services is outside the scope of practice of FDs and DPs, so their involvement is extremely low. . (“EVALUATION OF THE STRUCTURE AND PROVISION OF PRIMARY CARE IN TAJIKISTAN” 2014)
 
The poor integration of primary care and higher levels of care is another factor undermining continuity and quality of care. There is little follow-up for patients after specialist care or hospital treatment and limited exchange of information to allow primary care providers to carry on treatment and clinical management. In only 2 of 17 facilities covered by the study of rural health care providers were staff permanently available for treatment and referral (24 hours a day, 7 days a week) (Wiegers, Boerma & de Haan, 2011). . (Khodjamurodov et al. 2016)	
25	Weak performance on the attribute of comprehensiveness.	Documentation_required	COP1. Comprehensiveness of GP services 	A basic benefit package was adopted in principle in 2007, with the aim of defining which services should be provided at no cost (focused on essential primary and emergency care) and formalizing additional payments for others (as opposed to current informal payments). However, it is still in pilot mode and has so far only been extended to 14 of the country's 65 districts. .  (Khodjamurodov et al. 2016)	DES1. Referral system , DGN1. Laboratory , DGN2. Imaging, ORG4. Scope of practice of practitioners, SEL1. Preventive care, SEL2. Diagnostic procedures, SEL3. Treatment , SEL4. Management of diseases, SEL5. Paralell services, TCH1. Basic technology, WRK1. PC workforce planning	FDs clearly have access to better equipment than DPs and nurses. Marked differences in the equipment available to FDs and DPs are seen regionally. Availability of laboratory services and X-ray facilities is poor: one third of FDs and DPs have no or insufficient access to microbiological laboratory services and one quarter no or insufficient access to X-ray. Observed interregional differences are wide.  . (“EVALUATION OF THE STRUCTURE AND PROVISION OF PRIMARY CARE IN TAJIKISTAN” 2014)
 
FDs and DPs have a limited scope of practice, with a narrow range of curative services offered. Their role in first contact with patients' health problems is modest, as is their involvement in the treatment of diseases, although FDs are involved to a greater extent than DPs. Provision of medical procedures and preventive services is outside the scope of practice of FDs and DPs, so their involvement is extremely low. FDs and DPs are nevertheless very active in some domains, such as mother and child health care, reproductive health and some screening initiatives. Nurses are also active in mother and child health care and reproductive health. Most FDs and DPs are involved in tuberculosis health services through providing information, monitoring at-risk groups or providing treatment. Involvement in these tasks is more limited for nurses. . (“EVALUATION OF THE STRUCTURE AND PROVISION OF PRIMARY CARE IN TAJIKISTAN” 2014)
 
Gatekeeping by family medicine specialists at the primary level continues to be weak. Many patients access higher levels of care directly without referral from primary care and there is a need to strengthen systems of referral and coordination. There is also poor integration of primary and secondary care with regards to the continuity of care. . (“Review of the National Programme on the Development of Family Medicine 2011-2015 in Tajikistan” 2016)
 
FDs and DPs frequently provide referral letters to narrow specialists when patients are referred, but reporting back from specialists is weak. . (“EVALUATION OF THE STRUCTURE AND PROVISION OF PRIMARY CARE IN TAJIKISTAN” 2014)
 
FDs and DPs have a limited scope of practice, with a narrow range of curative services offered. Their role in first contact with patients' health problems is modest, as is their involvement in the treatment of diseases, although FDs are involved to a greater extent than DPs. Provision of medical procedures and preventive services is outside the scope of practice of FDs and DPs, so their involvement is extremely low. . (“EVALUATION OF THE STRUCTURE AND PROVISION OF PRIMARY CARE IN TAJIKISTAN” 2014)
 
In only 2 of 17 facilities covered by the study of rural health care providers were staff permanently available for treatment and referral (24 hours a day, 7 days a week) (Wiegers, Boerma & de Haan, 2011). . (Khodjamurodov et al. 2016)	
26	Weak performance on the attribute of coordination.	Documentation_required	COR1. Discharge management, COR2. Transition management	The MOH has defined family medicine doctors as the gatekeeper to health services in Tajikistan, according to the National Health Strategy 2010-2020. To realize this policy, under the BBP a new conditionality was placed on hospitalization: if a patient is referred for hospitalization by a family medicine doctor, then the patient will pay only a co-payment; whereas visiting hospitals directly means services are to be paid in full by the patient. This policy acts as an incentive for people to go to PHC and utilize services there rather than going directly to inpatient care. . (“Review of the National Programme on the Development of Family Medicine 2011-2015 in Tajikistan” 2016)
 
As part of the basic benefit package introduced in 2007, eight co-payment categories were created in the pilot rayons covered by the programme. For each category, the average amount a patient was supposed to contribute was set significantly lower than that reported for under-the-table payments for the same health care intervention. In addition, a 30% (for patients referred from the primary health care level) and 70% (for self-admission without any referral) co-payment differential was introduced. This differential co-payment was intended to strengthen the role of primary health care and to direct the flow of patients to primary health care units rather than hospitals. In 2009, co-payment levels increased to 50% and 80%, respectively. . (Khodjamurodov et al. 2016)	DES1. Referral system , FIN3. Financial protection in PC, FIN4. Comprehensiveness of financial protection for PC services	In 2014, the Health Policy and Analysis Unit (HPAU) carried out a study on access to PHC as part of an analysis of bottlenecks to health system strengthening (Akkazieva et al. 2015). With the introduction of family medicine as a gatekeeper to health care services, the referral system has been improved slightly and reinforced further by the introduction of the BBP. . (“Review of the National Programme on the Development of Family Medicine 2011-2015 in Tajikistan” 2016)
 
 
FDs and DPs usually work with several other physicians in the same premises; working solo in a primary care facility is rare for doctors. Many nurses work in so-called health houses without physician support. Health houses are structural units of rural health centres with a staff complement of one family nurse and one midwife per 750 population. They provide treatments prescribed by doctors and under medical guidance and supervision. In contrast to DPs, FDs usually work with nurses and have undertaken specialist training in family medicine. Most FDs and DPs regularly meet with other medical and nursing staff, but FDs do so more often. Most FDs (84.5%) and DPs (90.4%) regularly ask for advice from medical specialists. FDs and DPs' referral rates seem low, but variation within both groups is considerable. FDs have more frequent connections with community representatives. . (“EVALUATION OF THE STRUCTURE AND PROVISION OF PRIMARY CARE IN TAJIKISTAN” 2014)
 
Gatekeeping by family medicine specialists at the primary level continues to be weak. Many patients access higher levels of care directly without referral from primary care and there is a need to strengthen systems of referral and coordination. There is also poor integration of primary and secondary care with regards to the continuity of care. . (“EVALUATION OF THE STRUCTURE AND PROVISION OF PRIMARY CARE IN TAJIKISTAN” 2014)
 
FDs and DPs frequently provide referral letters to narrow specialists when patients are referred, but reporting back from specialists is weak. . (“EVALUATION OF THE STRUCTURE AND PROVISION OF PRIMARY CARE IN TAJIKISTAN” 2014)
 
The poor integration of primary care and higher levels of care is another factor undermining continuity and quality of care. There is little follow-up for patients after specialist care or hospital treatment and limited exchange of information to allow primary care providers to carry on treatment and clinical management. In only 2 of 17 facilities covered by the study of rural health care providers were staff permanently available for treatment and referral (24 hours a day, 7 days a week) (Wiegers, Boerma & de Haan, 2011). . (Khodjamurodov et al. 2016)	
27	Weak performance on the attribute of continuity.	Documentation_required	CON2. Follow-up care , CON2. Longitudinal continuity of care 	The MOH has defined family medicine doctors as the gatekeeper to health services in Tajikistan, according to the National Health Strategy 2010-2020. To realize this policy, under the BBP a new conditionality was placed on hospitalization: if a patient is referred for hospitalization by a family medicine doctor, then the patient will pay only a co-payment; whereas visiting hospitals directly means services are to be paid in full by the patient. This policy acts as an incentive for people to go to PHC and utilize services there rather than going directly to inpatient care. . (“Review of the National Programme on the Development of Family Medicine 2011-2015 in Tajikistan” 2016)
 
As part of the basic benefit package introduced in 2007, eight co-payment categories were created in the pilot rayons covered by the programme. For each category, the average amount a patient was supposed to contribute was set significantly lower than that reported for under-the-table payments for the same health care intervention. In addition, a 30% (for patients referred from the primary health care level) and 70% (for self-admission without any referral) co-payment differential was introduced. This differential co-payment was intended to strengthen the role of primary health care and to direct the flow of patients to primary health care units rather than hospitals. In 2009, co-payment levels increased to 50% and 80%, respectively. . (Khodjamurodov et al. 2016)	DES1. Referral system , FIN3. Financial protection in PC, FIN4. Comprehensiveness of financial protection for PC services	In 2014, the Health Policy and Analysis Unit (HPAU) carried out a study on access to PHC as part of an analysis of bottlenecks to health system strengthening (Akkazieva et al. 2015). With the introduction of family medicine as a gatekeeper to health care services, the referral system has been improved slightly and reinforced further by the introduction of the BBP. . (“Review of the National Programme on the Development of Family Medicine 2011-2015 in Tajikistan” 2016)
 
 
Patients' clinical records are well kept by doctors and nurses in primary care, but are paper-based as a rule, as computers are not available. FDs and DPs frequently provide referral letters to narrow specialists when patients are referred, but reporting back from specialists is weak. Patients are generally satisfied with their FD or DP but are critical about medical equipment and doctors' responsiveness to personal problems and worries. . (“EVALUATION OF THE STRUCTURE AND PROVISION OF PRIMARY CARE IN TAJIKISTAN” 2014)
 
Gatekeeping by family medicine specialists at the primary level continues to be weak. Many patients access higher levels of care directly without referral from primary care and there is a need to strengthen systems of referral and coordination. There is also poor integration of primary and secondary care with regards to the continuity of care. . (“Review of the National Programme on the Development of Family Medicine 2011-2015 in Tajikistan” 2016)
 
FDs and DPs frequently provide referral letters to narrow specialists when patients are referred, but reporting back from specialists is weak. . (“EVALUATION OF THE STRUCTURE AND PROVISION OF PRIMARY CARE IN TAJIKISTAN” 2014)
 
FDs and DPs have a limited scope of practice, with a narrow range of curative services offered. Their role in first contact with patients' health problems is modest, as is their involvement in the treatment of diseases, although FDs are involved to a greater extent than DPs. Provision of medical procedures and preventive services is outside the scope of practice of FDs and DPs, so their involvement is extremely low. . (“EVALUATION OF THE STRUCTURE AND PROVISION OF PRIMARY CARE IN TAJIKISTAN” 2014)
 
The poor integration of primary care and higher levels of care is another factor undermining continuity and quality of care. There is little follow-up for patients after specialist care or hospital treatment and limited exchange of information to allow primary care providers to carry on treatment and clinical management. In only 2 of 17 facilities covered by the study of rural health care providers were staff permanently available for treatment and referral (24 hours a day, 7 days a week) (Wiegers, Boerma & de Haan, 2011). . (Khodjamurodov et al. 2016)	
28	Weak and poorly coordinated vertical communicable disease programs.	Tajikistan has reported among the highest TB-burdened countries in the WHO European Region, with growing concern in recent years for increasing rates of multidrug-resistant TB (MDR-TB). Suboptimal delivery of TB services has been reasoned as a key contributing factor for poor health outcomes, with challenges including the concentration of TB treatment in hospitals, poor coordination between hospitals and primary care and weak monitoring systems. . (“Review of the National Programme on the Development of Family Medicine 2011-2015 in Tajikistan” 2016)	EFF1. Effective management and control of diseases	In recent years, Tajikistan has seen a significant volume of activity and commitment from the highest level of government for improvements in priority health areas. This includes prevention and control of communicable diseases, including preventing and reducing prevalence of viral hepatitis and waterborne infections . Clinical protocols for infectious diseases and parasitology for family medicine promote the crucial role of PHC, working closely with the population, in raising awareness on the importance of safe drinking water at home and the prevention and management of waterborne diseases. A number of achievements have supported the integration of HIV treatment and management into PHC during this period. In the process of developing CGPs described, HIV guidelines for PHC have been introduced. These guidelines reflect a comprehensive package of services for injection drug users, men who have sex with men (MSM), and sex workers. In-service training modules on HIV clinical care and prevention services have been developed based on these guidelines and integrated into postgraduate education. Pilot projects with Development Partners and NGOs have also worked to strengthen the resources available in family medicine centres and strengthen linkages between providers, NGOs, patients and the wider community. Prevention of vaccine-preventable diseases. Throughout the course of the Family Medicine Programme, PHC centres have conducted national vaccination dates against priority vaccine-preventable diseases in children.  High coverage of vaccination in accordance with regulated vaccine schedules has led to dramatic decreases in the incidence of diseases and elimination of mortality from vaccine-preventable diseases. Family medicine practice can be credited in large part with reductions in vaccine-preventable mortality as the delivery of vaccines is managed through the channels of family medicine. Prioritizing integrated, PHC-led TB care. In PHC, efforts have focused on strengthening the integration of Directly Observed Treatment Short course (DOTS). With support of Development Partners, this has included on-the-job training to improve monitoring and evaluation using a supportive supervision approach. Doing so has demonstrated improved coordination between vertical TB systems and PHC-level health workers providing the majority of services for TB patients. Coordination efforts have also focused on improving the linkages between PHC and inpatient facilities, improving collaboration between TB specialists and family medicine doctors by introducing collaborative TB-specialists to support PHC providers and share experiences . Responding to rates of TB and MDR-TB, Tajikistan's national TB programmes have consecutively worked to strengthen the integration of TB services in primary care. Over 3000 health providers have been trained on providing TB care in primary settings and accredited trainings are now in place to support these annually. The MOH and Ministry of Education have also worked to adapt the initial training and education of health workers for optimal TB service provision from the outset. Other cross-ministry partnerships have been established and a national committee brings together government officials from each ministry to collaborate on reducing TB. . (“Review of the National Programme on the Development of Family Medicine 2011-2015 in Tajikistan” 2016)
 
Strengthening TB services has been a high priority for the Government of Tajikistan since the approval of the first National TB Programme 1996–2000, which was then followed by the Concept of Public Health Reform and the National TB Programme 2003–2010. Guided by these programmes, the role of the primary care sector in TB service delivery was strengthened and full coverage of Directly Observed Treatment, Short-Course (DOTS) was achieved. The National TB Programme 2010–2015 was approved by the government to continue building on these successes. The National TB Programme 2010–2015 was designed to systematically address persisting TB service delivery gaps and further expand the role of primary care. Reorganizing the delivery of services under this Programme has allowed the majority of TB patients to start receiving TB care in outpatient facilities. Over 3000 health workers have been trained on providing TB services in primary care settings and annual accredited trainings are now in place to ensure these competencies are maintained. The Ministry of Health and Ministry of Education have worked to adapt the formal education of health workers to support the optimal provision of TB services and TB education has also been incorporated into the school system to increase population health literacy on TB. . (Bobokhojaev 2016)	DGN1. Laboratory , EFF1. Effective management and control of diseases, SEL1. Preventive care, SEL2. Diagnostic procedures, SEL3. Treatment , SEL4. Management of diseases, TCH1. Basic technology	Strategic documents of the Ministry of Health and Social Protection place emphasis on priority programmes and human capacity development in the areas of maternal and child health, HIV/AIDS, tuberculosis, malaria, polio and measles. There is little coordination across priority programmes and there is no consolidated human resource plan across priority programmes. Human resource development in Tajikistan is assisted by a number of international agencies and NGOs, but there are few formal mechanisms for aid coordination. . (Khodjamurodov et al. 2016)
 
Improved prevention and control of hepatitis. In recent years, reported cases of viral hepatitis and waterborne infections have decreased, dropping from 10 281 cases in 2013 to 5524 cases in 2014 (MOH 2014b). There has been progress in recent years on the reduction of TB incidence and mortality. Incidence of TB nationally in 2014 was 4.3 per 100 000 population, down from 6.2 in 2010 (MOH 2014b), with a decrease reported in nearly all oblasts. In 2013, nearly half (45.3%) of MDR-TB patients were treated as out-patients from the first day of treatment (Ahmedov et al. 2013). Reorganizing services delivery has allowed more than 70% of TB patients to now receive services through outpatient facilities. . (“Review of the National Programme on the Development of Family Medicine 2011-2015 in Tajikistan” 2016)	
29	Weak and poorly integrated non-communicable disease programs.	Documentation_required	EFF1. Effective management and control of diseases	In recent years, Tajikistan has seen a significant volume of activity and commitment from the highest level of government for improvements in priority health areas. This  includes, most recently, the national action plan on the control and prevention of NCDs for the period 2015-2018 . (“Review of the National Programme on the Development of Family Medicine 2011-2015 in Tajikistan” 2016)
 
 As part of the integration of NCDs in PHC, focus has been put on disease prevention and management and a package of essential interventions for NCDs in primary care (PEN)has been implemented. Towards the reduced incidence of NCDs, joint work plans were developed between the Family Medicine Centre, the Research and Clinical Oncology Centre and the Republican Cardiology Centre. This partnership has contributed to the development of guidelines for family doctors on the prevention of cardiovascular diseases, heart electrocardiograms,cervical cancer prevention, and breast cancer prevention. Other activities for hypertension included the development of evidence-based CGPs, CME modules and training materials. NCDs were also included in facility-based quality improvement activities such as active screening for hypertension, determining quality improvement indicators, regular feedback meetings and improving lab services. Patient support groups (hypertension and diabetes schools) have also been introduced to support adherence to treatment. . (“Review of the National Programme on the Development of Family Medicine 2011-2015 in Tajikistan” 2016)
 
In 2013, backed by the government's priority weighted to the prevention and control of NCDs, efforts to introduce WHO's package of essential NCD interventions (PEN) in primary care were initiated in Tajikistan. Constraints for the effective delivery of prevention measures in PHC, namely a lack of human resource capacity (high staff turnover, low qualifications), lack of material resources (diagnostic and laboratory equipment), and sub-standard methods (outdated CGPs, lack of screening methodologies), were key drivers for the introduction of the PEN framework. Initial piloting of PEN aimed to introduce primary prevention measures for heart attacks and strokes and secondary prevention after myocardial infarction, stroke and rheumatic heart disease. In 2013, a multidisciplinary working group was established with the objective of adapting and adjusting the PEN framework for implementation in Tajikistan; specifically, developing CGPs for PEN one, two and three and a training curriculum. Trainings for practicing providers were organized with EBMC in 2014, offering training-of-trainers in Dushanbe for workshops to then be provided at training centres in pilot districts. Together with TSMU, the curriculum for undergraduates was reviewed and greater emphasis placed on NCD prevention (such as screening services). . (“Review of the National Programme on the Development of Family Medicine 2011-2015 in Tajikistan” 2016)	CON1. Treatment, CON2. Follow-up care , CON2. Longitudinal continuity of care , DGN1. Laboratory , DGN2. Imaging, FIN3. Financial protection in PC, FIN4. Comprehensiveness of financial protection for PC services, GOV4. Quality assurance mechanisms, IMP2. Practice level quality improvement mechanisms , SEL6. Patient engagement , TCH1. Basic technology, UTL2. preventive care, WRK4. Academic status of PC	Survey results of providers in PEN pilot districts signal satisfaction with trainings and revised CGPs, finding these practical and easily applied. However, a monitoring process conducted in 2015 found human resources are still a main constraint for effective prevention and control services for NCDs in PHC. Availability of basic resources and medicines were also found to remain inconsistent, with CVD medicines, for example, not universally available or unaffordable in certain rayons. Nevertheless, implementation of PEN protocols has proven successful for improving the capacity for effective NCD services in PHC and the expansion of protocols to include cancer and extension of the pilot to additional rayons is planned. In clarifying services for delivery in PEN, referral processes have been streamlined towards more effective coordination between primary and secondary care. . (“Review of the National Programme on the Development of Family Medicine 2011-2015 in Tajikistan” 2016)	
30	Weak sub-national role and function (decentralization issues).	Tajikistan's health system has evolved from the Soviet model of health care, with so far few structural changes. The Soviet-style health system was generally comprehensive, but highly centralized, underfinanced and inefficient. . (Khodjamurodov et al. 2016)
The Tajik health system remains largely state owned and administered and the structure is generally (though not universally) hierarchical. However, the coordination between the national level, the oblast and rayon administrations and local health facilities is compromised by unclear accountability arrangements (such as those arising from the abolition of rayon-level health departments in 2012) and the absence of a formal mandate and authority for managing localized services. . (Khodjamurodov et al. 2016)
Some limited policy and administrative powers have been delegated from the national government to oblast administrations through the Law “on local administration and economy” of 1991 and the Law “on local government” of 1994. These laws allow oblasts to develop local health policies in line with the directives issued by the Ministry of Health and Social Protection and to allocate resources accordingly. .. (Khodjamurodov et al. 2016)
Apart from its quality of universal access, the organisation of the Soviet (Tajik) health system ran counter to the principles of Primary Health Care. The system was hierarchical and centralised, resources were concentrated on treatment at hospitals rather than prevention or outpatient treatment, and the workforce was highly specialised, rather than making effective use of generalist physicians and nurses .  (Parfitt and Cornish 2007)
Institutional harmonization for clear lines of accountability between the national level and regional and local facilities. Following the abolishment of rayon-level health departments in 2012, there is a critical need at present for a subnational coordinating mechanism and clarified roles and responsibilities. As previously noted, in 2013 the MOH established the PHC Coordination Council, chaired by a deputy minister of health and comprised of representatives from the MOH, national institutions and Development Partners. While serving as a means to promote policy dialogue and coordination, this has not alleviated the need to redefine lines of accountability between levels of the health system. . (Khodjamurodov et al. 2016)	GOV2. Accountability arrangements, MAN1. PC staffing  , MAN2. PC facility budgets, MAN3. Strategic planning 	Documentation_required	NoSubDimension	Documentation_required	
31	Weak primary care budgeting mechanisms and capabilities.	Budgetary funds to the health sector from the central government are distributed by the Ministry of Finance to the oblast administrations (hukumats) and managed by the oblast and rayon finance departments. The oblast and rayon authorities and finance departments: approve expenditures for health from local state budgets and distribute state funds at the oblast and rayon level; finance oblast level health facilities; receive financial accounts and monitor the use of resources; and submit financial reports to the Department of Economy and Financial Relations under the Ministry of Health and Social Protection. The oblast health departments have direct managerial and financial responsibility for their specialized and tertiary health facilities, as well as for the procurement and distribution of medical supplies and equipment to subordinated facilities. They have very limited financial resources to assist health facilities in their respective oblast. Oblast administration budgets do not include funds for health, except for those health institutions that are under their direct subordination, but consolidated oblast budgets include planned health sector expenditures for rayons. An oblast health department has limited staff, mainly responsible for inspecting. . (Khodjamurodov et al. 2016)	MAN2. PC facility budgets	Documentation_required	MAN2. PC facility budgets	Documentation_required	
32	Weak management and business planning capacity in primary care.	Capacity for planning, managing and decision-making is, therefore, essential in all health facilities. Despite Tajikistan's decentralized structure, with regional authorities and managerial positions across health facilities, managerial capacity remains limited in practice. . (“Review of the National Programme on the Development of Family Medicine 2011-2015 in Tajikistan” 2016)	MAN2. PC facility budgets, MAN3. Strategic planning 	Since 2005, the Sino Project has implemented business planning in several rayons. In 2012, tools for improving this practice were developed and the concept of business planning was sharpened. The project aims to increase managerial capacity in health institutions, in particular PHC, to raise awareness on the appropriate use of resources and increase available information in an effort to encourage managerial decisions based on identified population health needs. Activities. Strengthening business planning in PHC has worked to provide a number of tools for facility managers, including information on developing budgets and prioritization of resources, calculating per capita plans, and support for monitoring activities to identify problems and respond accordingly. . (Khodjamurodov et al. 2016)	MAN2. PC facility budgets, MAN3. Strategic planning 	To-date, strengthening business planning has been successfully introduced in 140 rural health centres and PHC management teams in the rayons of Shahrinav, Tursunzade, Vose, Hamadoni, Rudaki and Faizabad. Heads of institutions report business planning has improved general awareness of resources, increased autonomy in setting priorities and improved business planning activities. In 2014, the MOH decided on the rollout of business planning across all PHC facilities (Order No. 243, 28 April 2014); a marked success towards the institutionalization of the project's activities. Lessons learned. In order to harmonize different activities across levels of the health system, local political engagement is very important for a successful partnership with PHC facilities. In order to increase accountability, measures to involve communities in business planning are needed. Improving the management of services at the district level requires both the capacity for management and the assignment of clear functions and tasks to effectively carryout this process. . (“Review of the National Programme on the Development of Family Medicine 2011-2015 in Tajikistan” 2016)	
33	Ineffective physician payment mechanisms and poor incentives for quality	Family medicine doctors in Tajikistan are paid a flat salary based on the regulated norms (basic wage rate), independent of the size of the population they cover. The basic wage rate was approximately 465 somoni (US$ 97) in 2013. Under these circumstances, there appeared to be low motivation among providers to deliver quality care and be engaged in time-consuming prevention services, including patient education. . (“Review of the National Programme on the Development of Family Medicine 2011-2015 in Tajikistan” 2016)	FIN2. Payment methods in PC 	In order to improve the coverage and quality of basic health services, especially for women and children, the Ministry of Health and Social Protection has been implementing the Health Services Improvement Project with the support of the World Bank since 2013. Under this project, the use of performance-based financing at the primary health care level is being piloted. . (Khodjamurodov et al. 2016)
 
Since 2013, new performance payment mechanisms have been piloted in PHC in Tajikistan to improve the coverage and quality of PHC services, particularly maternal and child health. Moreover, 2013 saw the introduction of capitation mechanisms, piloted in select PHC facilities, aimed as a mechanism for improving the quality of services delivered in family medicine. In 2015, capitation payment was put into effect countrywide by ministerial order. . (Khodjamurodov et al. 2016)
 
The allocation of funds to PHC providers based on full capitation (applies to the whole health facility budget) has been piloted since 2013 with the support of Development Partners. This was first piloted in Sughd oblast and is planned for rollout across the country in coming years. Furthermore, between 2014 and 2015, the budget formation for PHC centres was revised by applying a capitation formula. It is planned that, in 2017, the budget for PHC will be formed based on capitation and should be increased to allow improved access and quality in PHC. In parallel, a new incentive mechanism of results-based financing for PHC has been piloted since 2013. This mechanism should strengthen human resource capacity, infrastructure and technical resources in facilities, contributing to the quality of health services. . (“Review of the National Programme on the Development of Family Medicine 2011-2015 in Tajikistan” 2016)
 
Performance-based financing is one component of the Health Services Improvement Project funded by the World Bank. It is aimed at improving the coverage and quality of basic PHC services in rural health facilities and selected rayons through the introduction of financial incentives for PHC providers based on performance indicators. Activities. A set of approximately 14 indicators have been used to score performance attached to financial incentives to be paid quarterly. Indicators include, for example, maternal and child health targets, as well as a few on NCDs, such as taking blood pressure measurements. To date, performance-based financing has been introduced in all eight pilot Rayons. . (“Review of the National Programme on the Development of Family Medicine 2011-2015 in Tajikistan” 2016)	FIN2. Payment methods in PC 	The salary of family medicine doctors is now 10% higher than specialists working in hospitals, with gains in the average wage of family doctors recorded from 58 somoni in 2007 to 122 somoni in 2008 to 513 somoni in 2012 (MOH 2013). . (“Review of the National Programme on the Development of Family Medicine 2011-2015 in Tajikistan” 2016)
 
 
The average monthly salary of physicians increased from 58 somoni in 2007 (approximately US$ 17 at the time) to 788 somoni in 2013 (approximately US$ 165 at the time) (Egamov, Bogadyrova & Akkazieva,  2014c). The average monthly salary of mid-level medical staff amounted to 489 somoni (approximately US$ 103 at the time) in 2013, while the average for junior-level medical staff amounted to 289 somoni (approximately US$ 61 at the time) (HPAU, 2013h). . (Khodjamurodov et al. 2016)
 
Being governmental employees, family doctors working at PHC level earn low wages. They were estimated to range between US$ 123 and US$ 153 per month in 2013 such that workers often rely on informal payments and in-kind contributions to earn additional Income . (Donadel et al. 2016)
 
One of the challenges is that the prestige of family medicine continues to be low. A survey among medical graduates in 2012 found that only 0.8% had chosen the specialty of family medicine, while 52.2% chose narrow specialties such as obstetrics/gynaecology or surgery and most preferred to work in hospitals in urban areas (HPAU, 2013e). Salary levels are one reason. While the salaries of health workers were increased by 40% in September 2012, the monthly salary of family doctors still only amounted to 513 somoni, which was slightly below the subsistence level of 536 somoni (approximately US$ 112) (HPAU, 2013e). Other challenges include poor working conditions and a lack of medical equipment (WHO Regional Office for Europe, 2014b). Those health workers who do work in family medicine might also face problems in using their working time productively. A qualitative study of 52 randomly selected health workers in family medicine (24 family doctors, 24 family nurses and 4 narrow specialists) from rayon and rural health centres in four rayons, conducted in July–August 2014, found that health workers spent a considerable time (41.1% of their working time over five consecutive days) on administrative tasks (in particular those related to the health information system), to the detriment of patient care (Bratschi et al., 2015). . (Khodjamurodov et al. 2016)
 
In Tajikistan, doctors at PHC level seem to overprescribe drugs, in view of the number of drugs prescribed concomitantly and the high level of polypharmacy. It can be explained by the low official salary FD earn from the government, estimated to range between US$ 123 and US$ 153 per month in 2013 which is not enough to cover essential needs. As a consequence, doctors often rely on informal payments and in-kind contributions from patients. Expenditures for drugs represented 2.8 % of governmental health budget in 2013 so that pharmaceuticals are mainly financed by patients through informal OPE (both at hospital and primary care level). Such conditions do not favour rational prescribing as the prescription of a high number of drugs can represent a complementary income source to doctors and pharmacists.. (Donadel et al. 2016)
 
Informal payments are not apprehended in this paper as a problem of corrupt practices, but as a symptom of the general condition of the Tajik health system that is under-financed and that lacks efficient and strong policy Instruments. The findings of this analysis show that the overall expenditures of primary health care patients in four study districts in Tajikistan, adjusted for inflation, have doubled between 2005 and 2011. This observation is mainly explained by an increase in the expenditures on medicine. In fact, not only do patients spend more money on prescribed drugs, but they are being prescribed and they obtain their medicine in larger proportions. . (Schwarz et al. 2013)
 
In a context where OPE are important, drugs represent an important income source for health service providers. Such a situation does not favour rational prescribing nor efficient service delivery, and is potentially harmful for patients. In particular, the economic ramifications cause high levels of expenditure for patients and households with detrimental, knock-on effects in the more vulnerable segments of the population. Analysis of the survey we conducted in 2014 in rural and semi-urban Tajikistan showed a high rate of drug prescription, an irrational use of antibiotics and vitamins and the common use of injections to administer medicines at the PHC level. Expenditures for drugs represented more than three-quarters of the total amount paid in the course of a visit to a PHC provider. It resulted that a third of the interviewees who did not obtain the drugs prescribed explained it to their inability to pay .  (Donadel et al. 2016)
 
The process of implementing performance-based payment was examined by two rounds of internal verification. As a result of its introduction, a significant difference in the amount of payments made to PHC providers between first and second quarters could be observed. During nine months of 2015, pilot PHC facilities earned 2 328 547 somoni, from which 70% was allocated as additional salary of health workers and the remaining 30% for the purpose of improving infrastructure. In terms of coverage, data on from the second quarter of 2015 showed that pilot facilities delivered 20% more PHC health services in comparison with the previous quarter. An assessment of quality indicators in the reporting period showed a 13% boost in the quality of services provided in pilot rayons in Sughd and 10% in Khatlon. . (“Review of the National Programme on the Development of Family Medicine 2011-2015 in Tajikistan” 2016)	
34	Low physician compensation	Family medicine doctors in Tajikistan are paid a flat salary based on the regulated norms (basic wage rate), independent of the size of the population they cover. The basic wage rate was approximately 465 somoni (US$ 97) in 2013. Under these circumstances, there appeared to be low motivation among providers to deliver quality care and be engaged in time-consuming prevention services, including patient education. . (“Review of the National Programme on the Development of Family Medicine 2011-2015 in Tajikistan” 2016)	FIN2. Payment methods in PC 	In order to improve the coverage and quality of basic health services, especially for women and children, the Ministry of Health and Social Protection has been implementing the Health Services Improvement Project with the support of the World Bank since 2013. Under this project, the use of performance-based financing at the primary health care level is being piloted. . (Khodjamurodov et al. 2016)
 
Since 2013, new performance payment mechanisms have been piloted in PHC in Tajikistan to improve the coverage and quality of PHC services, particularly maternal and child health. Moreover, 2013 saw the introduction of capitation mechanisms, piloted in select PHC facilities, aimed as a mechanism for improving the quality of services delivered in family medicine. In 2015, capitation payment was put into effect countrywide by ministerial order. . (Khodjamurodov et al. 2016)
 
The allocation of funds to PHC providers based on full capitation (applies to the whole health facility budget) has been piloted since 2013 with the support of Development Partners. This was first piloted in Sughd oblast and is planned for rollout across the country in coming years. Furthermore, between 2014 and 2015, the budget formation for PHC centres was revised by applying a capitation formula. It is planned that, in 2017, the budget for PHC will be formed based on capitation and should be increased to allow improved access and quality in PHC. In parallel, a new incentive mechanism of results-based financing for PHC has been piloted since 2013. This mechanism should strengthen human resource capacity, infrastructure and technical resources in facilities, contributing to the quality of health services. . (“Review of the National Programme on the Development of Family Medicine 2011-2015 in Tajikistan” 2016)
 
Performance-based financing is one component of the Health Services Improvement Project funded by the World Bank. It is aimed at improving the coverage and quality of basic PHC services in rural health facilities and selected rayons through the introduction of financial incentives for PHC providers based on performance indicators. Activities. A set of approximately 14 indicators have been used to score performance attached to financial incentives to be paid quarterly. Indicators include, for example, maternal and child health targets, as well as a few on NCDs, such as taking blood pressure measurements. To date, performance-based financing has been introduced in all eight pilot Rayons. . (“Review of the National Programme on the Development of Family Medicine 2011-2015 in Tajikistan” 2016)	FIN2. Payment methods in PC 	The salary of family medicine doctors is now 10% higher than specialists working in hospitals, with gains in the average wage of family doctors recorded from 58 somoni in 2007 to 122 somoni in 2008 to 513 somoni in 2012 (MOH 2013). . (“Review of the National Programme on the Development of Family Medicine 2011-2015 in Tajikistan” 2016)
 
 
The average monthly salary of physicians increased from 58 somoni in 2007 (approximately US$ 17 at the time) to 788 somoni in 2013 (approximately US$ 165 at the time) (Egamov, Bogadyrova & Akkazieva,  2014c). The average monthly salary of mid-level medical staff amounted to 489 somoni (approximately US$ 103 at the time) in 2013, while the average for junior-level medical staff amounted to 289 somoni (approximately US$ 61 at the time) (HPAU, 2013h). . (Khodjamurodov et al. 2016)
 
Being governmental employees, family doctors working at PHC level earn low wages. They were estimated to range between US$ 123 and US$ 153 per month in 2013 such that workers often rely on informal payments and in-kind contributions to earn additional Income . (Donadel et al. 2016)
 
One of the challenges is that the prestige of family medicine continues to be low. A survey among medical graduates in 2012 found that only 0.8% had chosen the specialty of family medicine, while 52.2% chose narrow specialties such as obstetrics/gynaecology or surgery and most preferred to work in hospitals in urban areas (HPAU, 2013e). Salary levels are one reason. While the salaries of health workers were increased by 40% in September 2012, the monthly salary of family doctors still only amounted to 513 somoni, which was slightly below the subsistence level of 536 somoni (approximately US$ 112) (HPAU, 2013e). Other challenges include poor working conditions and a lack of medical equipment (WHO Regional Office for Europe, 2014b). Those health workers who do work in family medicine might also face problems in using their working time productively. A qualitative study of 52 randomly selected health workers in family medicine (24 family doctors, 24 family nurses and 4 narrow specialists) from rayon and rural health centres in four rayons, conducted in July–August 2014, found that health workers spent a considerable time (41.1% of their working time over five consecutive days) on administrative tasks (in particular those related to the health information system), to the detriment of patient care (Bratschi et al., 2015). . (Khodjamurodov et al. 2016)
 
In Tajikistan, doctors at PHC level seem to overprescribe drugs, in view of the number of drugs prescribed concomitantly and the high level of polypharmacy. It can be explained by the low official salary FD earn from the government, estimated to range between US$ 123 and US$ 153 per month in 2013 which is not enough to cover essential needs. As a consequence, doctors often rely on informal payments and in-kind contributions from patients. Expenditures for drugs represented 2.8 % of governmental health budget in 2013 so that pharmaceuticals are mainly financed by patients through informal OPE (both at hospital and primary care level). Such conditions do not favour rational prescribing as the prescription of a high number of drugs can represent a complementary income source to doctors and pharmacists.. (Donadel et al. 2016)
 
Informal payments are not apprehended in this paper as a problem of corrupt practices, but as a symptom of the general condition of the Tajik health system that is under-financed and that lacks efficient and strong policy Instruments. The findings of this analysis show that the overall expenditures of primary health care patients in four study districts in Tajikistan, adjusted for inflation, have doubled between 2005 and 2011. This observation is mainly explained by an increase in the expenditures on medicine. In fact, not only do patients spend more money on prescribed drugs, but they are being prescribed and they obtain their medicine in larger proportions. . (Schwarz et al. 2013)
 
In a context where OPE are important, drugs represent an important income source for health service providers. Such a situation does not favour rational prescribing nor efficient service delivery, and is potentially harmful for patients. In particular, the economic ramifications cause high levels of expenditure for patients and households with detrimental, knock-on effects in the more vulnerable segments of the population. Analysis of the survey we conducted in 2014 in rural and semi-urban Tajikistan showed a high rate of drug prescription, an irrational use of antibiotics and vitamins and the common use of injections to administer medicines at the PHC level. Expenditures for drugs represented more than three-quarters of the total amount paid in the course of a visit to a PHC provider. It resulted that a third of the interviewees who did not obtain the drugs prescribed explained it to their inability to pay .  (Donadel et al. 2016)
 
The process of implementing performance-based payment was examined by two rounds of internal verification. As a result of its introduction, a significant difference in the amount of payments made to PHC providers between first and second quarters could be observed. During nine months of 2015, pilot PHC facilities earned 2 328 547 somoni, from which 70% was allocated as additional salary of health workers and the remaining 30% for the purpose of improving infrastructure. In terms of coverage, data on from the second quarter of 2015 showed that pilot facilities delivered 20% more PHC health services in comparison with the previous quarter. An assessment of quality indicators in the reporting period showed a 13% boost in the quality of services provided in pilot rayons in Sughd and 10% in Khatlon. . (“Review of the National Programme on the Development of Family Medicine 2011-2015 in Tajikistan” 2016)	
35	Low numbers of health care providers	The number of health workers, by comparison, has fallen precipitously since independence. From similar levels to EU averages at independence, there are now only 170 physicians per 100 000 population in Tajikistan (compared with 347 for the EU) and only 444 nurses per 100 000 (compared with 850 in the EU). . (Khodjamurodov et al. 2016)
 
Lack of an updated workforce registry (expert input)	WRK1. PC workforce planning, WRK3. PC workforce availability	National Programme on the Development of Family Medicine for the period 2011–2015 (MOH 2010a) [hereafter, Family Medicine Programme]. . (“Review of the National Programme on the Development of Family Medicine 2011-2015 in Tajikistan” 2016)
 
Development of a family medicine workforce is active across the country, with an increasing number of family doctors and nurses achieved in recent years. According to MOH data, there were 2338 family medicine doctors and 4416 family medicine nurses in 2014; equivalent to 28.3 per 100 000 population and 52.9 per 100 000 population, respectively (MOH 2014b). That same year, 48.1% of the total health workforce was working at the PHC level (MOH 2014b). Trends in the number of family medicine doctors and nurses per 100 000 population are reported in Table 11, showing a steady increase in recent years . (“Review of the National Programme on the Development of Family Medicine 2011-2015 in Tajikistan” 2016)
 
The Ministry of Health and Social Protection has introduced a range of incentives to improve the distribution and motivation of the health workforce. For example, it has used the allocation of land plots as a nonfinancial incentive to entice recent graduates to rural and remote areas. The Ministry has also adopted a policy that obliges recent graduates to spend the first three years after obtaining their diploma in rural areas (WHO Regional Office for Europe, 2014b), although in practice this policy has not been implemented. . (Khodjamurodov et al. 2016)
 
As there is a high awareness of geographical imbalances in the distribution of physicians, students  from underserved regions are favoured for medical school admission, but imbalances nevertheless remain. . (Khodjamurodov et al. 2016)
 
Feldsher (doctors' assistant) training was upgraded in 1996 to a four-year course in medical colleges. Feldshers work mainly in rural areas and fulfil an important function in the absence of physicians in these areas. . (Khodjamurodov et al. 2016)
 
Tajikistan's Ministry of Health now aims to reduce the numbers of physicians, and increase the numbers of nurses, to arrive at a ratio of 1:6 (European Observatory on Health Care Systems, 2000). In order to enable nurses to take on greater clinical responsibilities, their level of education and skills are also being Increased. . (Parfitt and Cornish 2007)	FIN1. Primary care expenditure , FIN2. Payment methods in PC , GOV1. Primary care priorities , GOV3. Stakeholder participation and engagement, GOV4. Quality assurance mechanisms, WRK1. PC workforce planning, WRK2. Financial status of GPs/FMs, WRK3. PC workforce availability, WRK4. Academic status of PC	There are estimated to be 27.6 GPs per 100,000 population (2013). Physicians are concentrated in the capital, Dushanbe, while the density of all staff categories (except feldshers) is lowest in Khatlon oblast and the Districts of Republican Subordination. Challenges in rural and remote areas include poor human resource management, low salaries, outdated medical equipment and the poor condition of health facilities. . (Khodjamurodov et al. 2016)
 
A particularly drastic decline occurred in the number of midwives per 100 000 population, falling from 129 in 1990 to 54 in 2013 (WHO Regional Office for Europe, 2015a).. (Khodjamurodov et al. 2016)
 
Challenges in rural and remote areas include poor human resource management, low salaries, outdated medical equipment and the poor condition of health facilities. . (Khodjamurodov et al. 2016)
 
47% increase in # of FM doctors, and 74% increase in # of FM nurses between 2010-2014 . (“Review of the National Programme on the Development of Family Medicine 2011-2015 in Tajikistan” 2016)
 
Health workforce registration for planning and forecasting between pre- and postgraduate training. Policy dialogue to more tightly coordinate planning and development of human resources in the medical field is needed. Establishing the registration of health providers and a database on health workforce demographics (education, age and sex), employment status and composition, are important steps towards improved planning. . (“Review of the National Programme on the Development of Family Medicine 2011-2015 in Tajikistan” 2016)
 
Physician density in Tajikistan is relatively low. Official data report 15 973 active physicians in 2012, which equates to 205 physicians per 100 000 population. Only 11.5% are working in primary care, either as retrained family doctors(FDs) or district physicians / therapists (DPs). Numbers of FDs and nurses are increasing following the introduction of retraining programmes and a postgraduate programme in 2009, albeit at modest pace. The imbalance between rural and urban coverage and the high migration of skilled health staff. Shortage of staff in PHC, particularly FDs and nurses in rural areas, should be addressed, starting with a human resource plan. Official reports and the survey results point to the serious problem of staff shortages. . (“EVALUATION OF THE STRUCTURE AND PROVISION OF PRIMARY CARE IN TAJIKISTAN” 2014)	
36	Poorly distributed health care workforce	The number of health workers, by comparison, has fallen precipitously since independence. From similar levels to EU averages at independence, there are now only 170 physicians per 100 000 population in Tajikistan (compared with 347 for the EU) and only 444 nurses per 100 000 (compared with 850 in the EU). . (Khodjamurodov et al. 2016)
 
Lack of an updated workforce registry (expert input)	WRK1. PC workforce planning, WRK3. PC workforce availability	National Programme on the Development of Family Medicine for the period 2011–2015 (MOH 2010a) [hereafter, Family Medicine Programme]. . (“Review of the National Programme on the Development of Family Medicine 2011-2015 in Tajikistan” 2016)
 
Development of a family medicine workforce is active across the country, with an increasing number of family doctors and nurses achieved in recent years. According to MOH data, there were 2338 family medicine doctors and 4416 family medicine nurses in 2014; equivalent to 28.3 per 100 000 population and 52.9 per 100 000 population, respectively (MOH 2014b). That same year, 48.1% of the total health workforce was working at the PHC level (MOH 2014b). Trends in the number of family medicine doctors and nurses per 100 000 population are reported in Table 11, showing a steady increase in recent years . (“Review of the National Programme on the Development of Family Medicine 2011-2015 in Tajikistan” 2016)
 
The Ministry of Health and Social Protection has introduced a range of incentives to improve the distribution and motivation of the health workforce. For example, it has used the allocation of land plots as a nonfinancial incentive to entice recent graduates to rural and remote areas. The Ministry has also adopted a policy that obliges recent graduates to spend the first three years after obtaining their diploma in rural areas (WHO Regional Office for Europe, 2014b), although in practice this policy has not been implemented. . (Khodjamurodov et al. 2016)
 
As there is a high awareness of geographical imbalances in the distribution of physicians, students  from underserved regions are favoured for medical school admission, but imbalances nevertheless remain. . (Khodjamurodov et al. 2016)
 
Feldsher (doctors' assistant) training was upgraded in 1996 to a four-year course in medical colleges. Feldshers work mainly in rural areas and fulfil an important function in the absence of physicians in these areas. . (Khodjamurodov et al. 2016)
 
Tajikistan's Ministry of Health now aims to reduce the numbers of physicians, and increase the numbers of nurses, to arrive at a ratio of 1:6 (European Observatory on Health Care Systems, 2000). In order to enable nurses to take on greater clinical responsibilities, their level of education and skills are also being Increased. . (Parfitt and Cornish 2007)	FIN1. Primary care expenditure , FIN2. Payment methods in PC , GOV1. Primary care priorities , GOV3. Stakeholder participation and engagement, GOV4. Quality assurance mechanisms, WRK1. PC workforce planning, WRK2. Financial status of GPs/FMs, WRK3. PC workforce availability, WRK4. Academic status of PC	There are estimated to be 27.6 GPs per 100,000 population (2013). Physicians are concentrated in the capital, Dushanbe, while the density of all staff categories (except feldshers) is lowest in Khatlon oblast and the Districts of Republican Subordination. Challenges in rural and remote areas include poor human resource management, low salaries, outdated medical equipment and the poor condition of health facilities. . (Khodjamurodov et al. 2016)
 
A particularly drastic decline occurred in the number of midwives per 100 000 population, falling from 129 in 1990 to 54 in 2013 (WHO Regional Office for Europe, 2015a).. (Khodjamurodov et al. 2016)
 
Challenges in rural and remote areas include poor human resource management, low salaries, outdated medical equipment and the poor condition of health facilities. . (Khodjamurodov et al. 2016)
 
47% increase in # of FM doctors, and 74% increase in # of FM nurses between 2010-2014 . (“Review of the National Programme on the Development of Family Medicine 2011-2015 in Tajikistan” 2016)
 
Health workforce registration for planning and forecasting between pre- and postgraduate training. Policy dialogue to more tightly coordinate planning and development of human resources in the medical field is needed. Establishing the registration of health providers and a database on health workforce demographics (education, age and sex), employment status and composition, are important steps towards improved planning. . (“Review of the National Programme on the Development of Family Medicine 2011-2015 in Tajikistan” 2016)
 
Physician density in Tajikistan is relatively low. Official data report 15 973 active physicians in 2012, which equates to 205 physicians per 100 000 population. Only 11.5% are working in primary care, either as retrained family doctors(FDs) or district physicians / therapists (DPs). Numbers of FDs and nurses are increasing following the introduction of retraining programmes and a postgraduate programme in 2009, albeit at modest pace. The imbalance between rural and urban coverage and the high migration of skilled health staff. Shortage of staff in PHC, particularly FDs and nurses in rural areas, should be addressed, starting with a human resource plan. Official reports and the survey results point to the serious problem of staff shortages. . (“EVALUATION OF THE STRUCTURE AND PROVISION OF PRIMARY CARE IN TAJIKISTAN” 2014)	
