# Supplementary material for: A problem-oriented systems approach to primary care system development: development and initial testing of the problem-oriented primary care system development record
Source: BMC Health Serv Res. 2020 Aug 1;20:706. doi: 10.1186/s12913-020-05581-z (PMC7395390; doi:10.1186/s12913-020-05581-z)
Supplement: Supplementary file 5 — Additional file 5. Supplementary file containing the following: the full Table 1, Fig. 9 and Fig. 10. Table 1. Query of Tajikistan’s Problem Statement list and Problem System (Probα) codes. Fig. 9. Socioeconomic context table comprised of UNData indicators, by year (screenshot). Fig. 10. Fragility context table comprised of Deutsches Institut für Entwicklungspolitik (DIE) Constellations of State Fragility and Fragile State Index (FSI) indicators, by year (screenshot). [file 12913_2020_5581_MOESM5_ESM.docx]

**Supplementary file**

Table 1. Query of Tajikistan’s Problem Statement list and Problem System (Probα) codes.

| **ID** | **Problem_statement** | **Problem_WHOcode (Probα Code)** |
| --- | --- | --- |
| 1 | Weak policy and legal frameworks to reorient the health system towards primary care and family medicine. | GOV1. Primary care priorities |
| 2 | Weak and fragmented governance and accountability mechanisms related to the development and strengthening of primary care. | GOV2. Accountability arrangements |
| 3 | Weak public role in relation to the governance and/or organization of the primary care system. | GOV3. Stakeholder participation and engagement |
| 4 | Low Total Health Expenditure (THE) | FIN1. Primary care expenditure |
| 5 | Weak financing mechanisms – revenue collection, pooling, and coverage. | FIN3. Financial protection in PC, FIN4. Comprehensiveness of financial protection for PC services |
| 6 | Lack of a Basic Health Services Package (BHSP) / Basic Benefits Package. | FIN3. Financial protection in PC |
| 7 | High private, Out of Pocket (OOP) and informal expenditure, as a percent of Total Health Expenditure (THE). | FIN4. Comprehensiveness of financial protection for PC services |
| 8 | Poor health system allocative efficiency. | GOV2. Accountability arrangements |
| 9 | Weak funding mechanisms for primary care facilities. | FIN1. Primary care expenditure |
| 11 | Weak infrastructure and equipment. | DGN1. Laboratory, DGN2. Imaging, STR 1. Basic amenities, TCH1. Basic technology |
| 12 | Weak regulation and governance of pharmaceuticals. | MED1. Essential medicine list, MED2. Generic prescribing |
| 13 | Weak health information management systems. | INF1. Data capture, INF2. Aggregation of data |
| 15 | Outdated education and training standards for the specialty of family medicine and general practice. | WRK4. Academic status of PC |
| 16 | Weak and outdated standards for education, training and licensing of nurses in primary care. | WRK4. Academic status of PC |
| 17 | Weak Continuing Medical Education (CME) standards. | WRK4. Academic status of PC |
| 18 | The domain of primary care suffers low prestige and weak professional standards. | IMP5. Job satisfaction, WRK4. Academic status of PC |
| 19 | Weak recruitment and retention. | WRK3. PC workforce availability |
| 20 | Weak accreditation standards in primary care settings. | GOV4. Quality assurance mechanisms |
| 21 | Weak regulation of quality of care. | GOV4. Quality assurance mechanisms |
| 22 | Weak standards for the development of clinical guidelines and protocols for primary care settings. | GOV4. Quality assurance mechanisms |
| 23 | Weak implementation of clinical guidelines and standards in primary care settings. | GOV4. Quality assurance mechanisms |
| 24 | Weak performance on the attribute of first contact care. | ACS1. Accessibility, ACS2. Financial affordability, ACS3. Acceptability |
| 25 | Weak performance on the attribute of comprehensiveness. | COP1. Comprehensiveness of GP services |
| 26 | Weak performance on the attribute of coordination. | COR1. Discharge management, COR2. Transition management |
| 27 | Weak performance on the attribute of continuity. | CON2. Follow-up care, CON2. Longitudinal continuity of care |
| 28 | Weak and poorly coordinated vertical communicable disease programs. | EFF1. Effective management and control of diseases |
| 29 | Weak and poorly integrated non-communicable disease programs. | EFF1. Effective management and control of diseases |
| 30 | Weak sub-national role and function (decentralization issues). | GOV2. Accountability arrangements, MAN1. PC staffing, MAN2. PC facility budgets, MAN3. Strategic planning |
| 31 | Weak primary care budgeting mechanisms and capabilities. | MAN2. PC facility budgets |
| 32 | Weak management and business planning capacity in primary care. | MAN2. PC facility budgets, MAN3. Strategic planning |
| 33 | Ineffective physician payment mechanisms and poor incentives for quality | FIN2. Payment methods in PC |
| 34 | Low physician compensation | FIN2. Payment methods in PC |
| 35 | Low numbers of health care providers | WRK1. PC workforce planning, WRK3. PC workforce availability |
| 36 | Poorly distributed health care workforce | WRK1. PC workforce planning, WRK3. PC workforce availability |


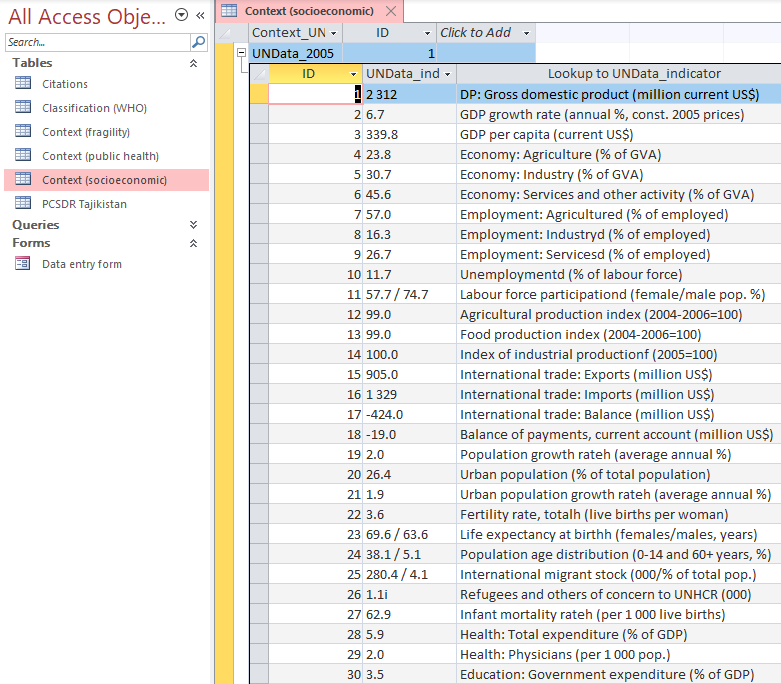


Fig. 9. Socioeconomic context table comprised of UNData indicators, by year (screenshot).


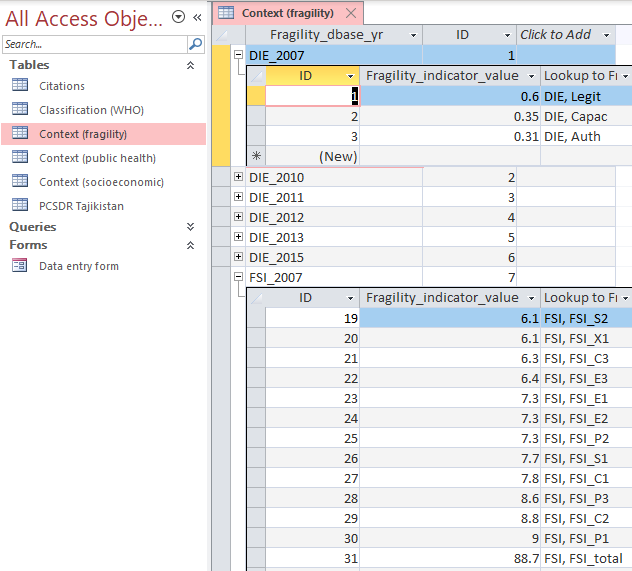
Fig. 10. Fragility context table comprised of Deutsches Institut für Entwicklungspolitik (DIE) Constellations of State Fragility and Fragile State Index (FSI) indicators, by year (screenshot).
